# Supplementary material for: Stimulating at the right time to recover network states in a model of the cortico-basal ganglia-thalamic circuit
Source: PLoS Comput Biol. Author manuscript; Available in PMC 2022 Mar 29. (PMC8939795; doi:10.1371/journal.pcbi.1009887)
Supplement: S1 Appendix [file EMS143856-supplement-S1_Appendix.docx]

## S1 Appendix: Extended Model Description

For regions where there is local coupling between populations, H_i,j_ parameterizes the synaptic gain from the *j^th^* to *i^th^* population (e.g., in cortical model which consists of four coupled populations). Unlike long distance connectivity (between-regions), delays between populations within each region are assumed to be short and can be modelled alongside membrane responses in terms of a lumped time constant *τ_i_.* All subcortical populations and the middle/deep layers of the motor cortex receive independent stochastic inputs *u_i_* modelling endogenous noise.

The neural mass model for each region is as follows:

*Cortex (M2)*

A middle layer composed of middle pyramidal cells with inhibitory self-connection (with strength parameterized by H_1,1_):

$\dot{v_{1}}=x_{1}$,

$\dot{x}_{1}=\frac{1}{\tau_{1}}\left( -H_{1,1} S_{1}\left( v_{1} \right)-H_{1,3} S_{1}\left( v_{3} \right)+H_{1,2} S_{1}\left( v_{2} \right)+A^{1} \right)-\frac{2}{\tau_{1}}x_{1}-\frac{1}{\tau_{1}^{2}}v_{1}$;

*Equation (S1.4)*

A supra-granular layer composed of superficial pyramidal cells with inhibitory self-connection (with strength parameterized by H_2,2_):

$\dot{v_{2}}=x_{2}$,

$\dot{x_{2}}=\frac{1}{\tau_{2}}\left( {-H}_{2,2} S_{2}\left( v_{2} \right)+H_{2,1} S_{2}\left( v_{1} \right)-H_{2,3} S_{2}\left( v_{3} \right)+H_{2,4} S_{2}\left( v_{4} \right)+u_{2} \right)-\frac{2}{\tau_{2}}x_{2}-\frac{1}{\tau_{2}^{2}}v_{2}$;

*Equation (S1.5)*

The supra-granular layer also contains a separate inhibitory interneuron population, again with an inhibitory self-connection (with strength parameterized by H_3,3_):

$\dot{v_{3}}=x_{3}$,

$\dot{x_{3}}=\frac{1}{\tau_{3}}\left( {-H}_{3,3} S_{3}\left( v_{3} \right)+H_{3,1} S_{3}\left( v_{1} \right)+H_{3,4} S_{3}\left( v_{4} \right)+H_{3,2} S_{3}\left( v_{2} \right) \right)-\frac{2}{\tau_{3}}x_{3}-\frac{1}{\tau_{3}^{2}}v_{3}$;

*Equation (S1.6)*

Finally, the infra-granular layer is made up of deep pyramidal cells also with an inhibitory self-connection (with strength parameterized by H_4,4_)

$\dot{v_{4}}=x_{4}$,

$\dot{x_{4}}=\frac{1}{\tau_{4}}\left( {-H}_{4,4} S_{4}\left( v_{4} \right)-H_{4,3} S_{4}\left( v_{3} \right)+H_{4,2} S_{4}\left( v_{2} \right)+u_{4} \right)-\frac{2}{\tau_{4}}x_{4}-\frac{1}{\tau_{4}^{2}}v_{4}$.

*Equation (S1.7)*

Overall, the cortical output is equal to the voltage in the deep pyramidal layer, thus:

$V^{1}=v_{4}$.

*Equation (S1.8)*

*Striatum (STR)*

Comprises single inhibitory population and self-inhibitory connection (with strength parameterized by H_5,5_):

$\dot{v_{5}}=x_{5}$,

$\dot{x_{5}}=\frac{1}{\tau_{5}}\left( {-H}_{5,5} S_{5}\left( v_{5} \right)+A^{2}+u_{5} \right)-\frac{2}{\tau_{5}}x_{5}-\frac{1}{\tau_{5}^{2}}v_{5}$;

*Equation (S1.9)*

and striatal output:

$V^{2}=v_{5}$.

*Equation (S1.10)*

*External part of globus pallidus (GPe)*

Comprises a single inhibitory population:

$\dot{v_{6}}=x_{6}$,

$\dot{x_{6}}=\frac{1}{\tau_{6}}\left( A^{3}+u_{6} \right)-\frac{2}{\tau_{6}}x_{6}-\frac{1}{\tau_{6}^{2}}v_{6}$,

*Equation (S1.11)*

and output from GPe:

$V^{3}=v_{6}$.

*Equation (S1.12)*

*Subthalamic nucleus (STN)*

Comprises a single excitatory population:

$\dot{v_{7}}=x_{7}$,

$\dot{x_{7}}=\frac{1}{\tau_{7}}\left( A^{4}+u_{7} \right)-\frac{2}{\tau_{7}}x_{7}-\frac{1}{\tau_{7}^{2}}v_{7}$,

*Equation (S1.13)*

and subthalamic output:

$V^{4}=v_{7}$.

*Equation (S1.14)*

*Internal segment of globus pallidus (GPi)*

Comprises a single inhibitory population:

$\dot{v_{8}}=x_{8}$,

$\dot{x_{8}}=\frac{1}{\tau_{8}}\left( A^{5}+u_{8} \right)-\frac{2}{\tau_{8}}x_{8}-\frac{1}{\tau_{8}^{2}}v_{8}$,

*Equation (S1.15)*

and output from GPi:

$V^{5}=v_{8}$.

*Equation (S1.16)*

*Thalamus (Thal.)*

Comprises a single excitatory population with self-inhibition (with strength parameterized by H_9,9_):

$\dot{v_{9}}=x_{9}$,

$\dot{x_{9}}=\frac{1}{\tau_{9}}\left( -H_{9,9}S_{9}\left( v_{9} \right)+A^{6}+u_{9} \right)-\frac{2}{\tau_{9}}x_{9}-\frac{1}{\tau_{9}^{2}}v_{9}$,

*Equation (S1.17)*

and thalamic output:

$V^{6}=v_{9}$.

*Equation (S1.18)*

Stochastic drive was given to all subcortical populations and the middle layer of the motor cortex.

The parameters of the model are given in the table below:

| **Table of Model Parameters** | | | | |  |
| --- | --- | --- | --- | --- | --- |
| **Cortex** | *Prior* | *Prior variance* | *Posterior -log scaling* | *Posterior* | *Ref*^[[1]](#footnote-1)^ |
| Time constant (*ms*) |  |  |  |  |  |
| $\tau_{1}:$*middle (mp)* | 3 | 1/4 | -0.48 | 2 | [1] |
| $\tau_{2}:$*superficial (sp)* | 2 | 1/4 | -0.27 | 2 |  |
| $\tau_{3}:$*interneurons (ii)* | 12 | 1/4 | 0.35 | 17 |  |
| $\tau_{4}:$*deep (dp)* | 18 | 1/4 | -0.19 | 15 |  |
| Synaptic gain (*Hz*)^[[2]](#footnote-2)^ |  |  |  |  |  |
| $H_{1,1}:$*mp → mp (self inh.)* | 400 | 1/4 | 0.15 | 464.90 | [2,3] |
| $H_{2,1}:$*mp → sp* | 800 | 1/4 | 0.41 | 1207.82 |  |
| $H_{1,3}:$*ii → mp* | 400 | 1/4 | 0.07 | 431.02 |  |
| $H_{3,3}:$*ii → ii (self inh.)* | 400 | 1/4 | 0.72 | 822.30 |  |
| $H_{3,1}:$*mp → ii* | 400 | 1/4 | -0.95 | 155.41 |  |
| $H_{3,4}:$*dp → ii* | 400 | 1/4 | 0.35 | 565.31 |  |
| $H_{2,2}:$*sp → sp (self inh.)* | 400 | 1/4 | 0.01 | 402.60 |  |
| $H_{1,2}:$*sp → mp* | 400 | 1/4 | 0.40 | 593.93 |  |
| $H_{4,3}:$*ii → dp* | 400 | 1/4 | -0.50 | 241.61 |  |
| $H_{4,4}:$*dp → dp (self inh.)* | 400 | 1/4 | 0.51 | 667.42 |  |
| $H_{4,2}:$*sp → dp* | 800 | 1/4 | -0.92 | 318.44 |  |
| $H_{2,3}:$*ii → sp* | 400 | 1/4 | 0.46 | 632.43 |  |
| $H_{3,2}:$*sp → ii* | 400 | 1/4 | 0.33 | 554.68 |  |
| $H_{2,4}:$*dp → sp* | 400 | 1/4 | -0.07 | 371.34 |  |
| Input gain (*scalar*) |  |  |  |  |  |
| $C_{1}:$*input to mp* | 1 | 1/16 | -0.066 | 0.9364 |  |
| $C_{4}:$*input to dp* | 1 | 1/16 | -0.066 | 0.9364 |  |
| **Striatum** | *Prior* | *Prior variance* | *Posterior -log scaling* | *Posterior* |  |
| Time constant (*ms*) |  |  |  |  |  |
| $\tau_{5}$ | 8 | 1/8 | -0.05 | 8 | [4] |
| Synaptic gain (*Hz*) |  |  |  |  |  |
| $H_{5,5}:$*self inh.* | 400 | 1/8 | 2.12 | 849 |  |
| Input gain (*scalar)* |  |  |  |  |  |
| $C_{5}$ | 1 | 1/16 | 0.25 | 1.288 |  |
| **GPe** | *Prior* | *Prior variance* | *Posterior -log scaling* | *Posterior* |  |
| Time constant (*ms*) |  |  |  |  |  |
| $\tau_{6}$ | 8 | 1/8 | 0.49 | 13 | [5] |
| Input gain (*scalar)* |  |  |  |  |  |
| $C_{6}$ | 1 | 1/16 | -0.207 | 0.813 |  |
| **STN** | *Prior* | *Prior variance* | *Posterior -log scaling* | *Posterior* |  |
| Time constant (*ms*) |  |  |  |  |  |
| $\tau_{7}$ | 4 | 1/8 | -0.68 | 2 | [6] |
| Input gain (*scalar*) |  |  |  |  |  |
| $C_{7}$ | 1 | 1/16 | -0.188 | 0.829 |  |
| **GPI** | *Prior* | *Prior variance* | *Posterior -log scaling* | *Posterior* |  |
| Time constant (*ms*) |  |  |  |  |  |
| $\tau_{8}$ | 8 | 1/8 | -0.01 | 8 | [5] |
| Input gain (*scalar*) |  |  |  |  |  |
| $C_{8}$ | 1 | 1/16 | -0.14 | 0.874 |  |
| **Thalamus** | *Prior* | *Prior variance* | *Posterior -log scaling* | *Posterior* |  |
| Time constant (*ms*) |  |  |  |  |  |
| $\tau_{9}$ | 8 | 1/8 | 1 | 8 | [7] |
| Synaptic gain (*Hz*) |  |  |  |  |  |
| $H_{9,9}:$*self inh.* | 400 | 1/8 | 1.82 | 729 |  |
| Input gain (*scalar*) |  |  |  |  |  |
| $C_{9}$ | 1 | 1/16 | 0.37 | 1.45 |  |
| **Coupling Parameters** | *Prior* | *Prior variance* | *Posterior -log scaling* | *Posterior* |  |
| Connection weights (*Hz*) |  |  |  |  |  |
| $\omega_{2,1}:$M2 → STR | 1600 | 1/4 | -0.22 | 1284 | [2,3] |
| $\omega_{4,1}:$M2 → STN | 2000 | 1/4 | -0.64 | 1055 |  |
| $\omega_{6,1}:$M2 → Thal | 1000 | 1/4 | 0.164 | 1178 |  |
| $\omega_{3,2}:$STR → GPe | -2000 | 1/4 | -0.17 | -1687 |  |
| $\omega_{5,2}:$STR → GPi | -1600 | 1/4 | 0.87 | -3819 |  |
| $\omega_{4,3}:$GPe → STN | -2000 | 1/4 | 0.9 | -4919 |  |
| $\omega_{3,4}:$STN → GPe | 2000 | 1/4 | -0.7 | 993 |  |
| $\omega_{5,4}:$STN → GPi | 1600 | 1/4 | -0.22 | 1284 |  |
| $\omega_{6,5}:$GPi → Thal | -1000 | 1/4 | 0.54 | -1716 |  |
| $\omega_{1,6}:$Thal → M2 | 2000 | 1/4 | 0.14 | 2301 |  |
| Delays (*ms*) |  |  |  |  |  |
| $D_{2,1}:$M2 → STR | 3 | 1/16 | 0.68 | 6 | [8] |
| $D_{4,1}:$M2 → STN | 3 | 1/16 | -0.03 | 3 |  |
| $D_{6,1}:$M2 → Thal. | 4 | 1/16 | 0.61 | 7.5 |  |
| $D_{3,2}:$STR → GPe | 7 | 1/16 | -0.44 | 5 |  |
| $D_{5,2}:$STR → GPi | 12 | 1/16 | -0.55 | 7 |  |
| $D_{4,3}:$GPe → STN | 1 | 1/16 | -0.58 | 1 |  |
| $D_{3,4}:$STN → GPe | 3 | 1/16 | -0.56 | 2 |  |
| $D_{5,4}:$STN → GPi | 3 | 1/16 | 0.39 | 4 |  |
| $D_{6,5}:$GPi → Thal | 3 | 1/16 | 0.56 | 5.5 |  |
| $D_{1,6}:$Thal → M2 | 8 | 1/16 | 0.65 | 15.5 |  |
|  |  |  |  |  |  |

Supporting References

1. Aerde KIV, Qi G, Feldmeyer D. Cell type-specific effects of adenosine on cortical neurons. Cereb Cortex. 2015;25: 772–787. doi:10.1093/cercor/bht274

2. Jansen BH, Rit VG. Electroencephalogram and visual evoked potential generation in a mathematical model of coupled cortical columns. Biol Cybern. 1995;73: 357–366. doi:10.1007/BF00199471

3. van Wijk BCM, Cagnan H, Litvak V, Kühn AA, Friston KJ. Generic dynamic causal modelling: An illustrative application to Parkinson’s disease. Neuroimage. 2018;181: 818–830. doi:10.1016/J.NEUROIMAGE.2018.08.039

4. Planert H, Berger TK, Silberberg G. Membrane Properties of Striatal Direct and Indirect Pathway Neurons in Mouse and Rat Slices and Their Modulation by Dopamine. Brown JT, editor. PLoS One. 2013;8: e57054. doi:10.1371/journal.pone.0057054

5. Nambu A, Llinas R. Electrophysiology of globus pallidus neurons in vitro. J Neurophysiol. 1994;72: 1127–1139. doi:10.1152/jn.1994.72.3.1127

6. Kita H, Chang HT, Kitai ST. Pallidal inputs to subthalamus: Intracellular analysis. Brain Res. 1983;264: 255–265. doi:10.1016/0006-8993(83)90823-5

7. Paz JT, Chavez M, Saillet S, Deniau JM, Charpier S. Activity of ventral medial thalamic neurons during absence seizures and modulation of cortical paroxysms by the nigrothalamic pathway. J Neurosci. 2007;27: 929–941. doi:10.1523/JNEUROSCI.4677-06.2007

8. Plenz D, Kital ST. A basal ganglia pacemaker formed by the subthalamic nucleus and external globus pallidus. Nature. 1999;400: 677–82. doi:10.1038/23281

1. References given to support the range and scale of chosen parameters; differences are likely to occur for studies investigating different species. These models are also so called “lumped” parameter models and so incorporate not just membrane time constants, but also factor in delays arising from synaptic transmission and dendritic propagation. [↑](#footnote-ref-1)
2. Synaptic gains are relatively abstract parameters that summarise the “average synaptic contacts” per population. Mostly the values follow from the original paper of [2], as well as the empirical fits provided by [3]. [↑](#footnote-ref-2)
