# Supplementary material for: Stimulating at the right time to recover network states in a model of the cortico-basal ganglia-thalamic circuit
Source: PLoS Comput Biol. Author manuscript; Available in PMC 2022 Mar 29. (PMC8939795; doi:10.1371/journal.pcbi.1009887)
Supplement: S1 Fig [file EMS143856-supplement-S1_Fig.docx]

## S1 Supplementary Figure – Full set of empirical data features and model fits


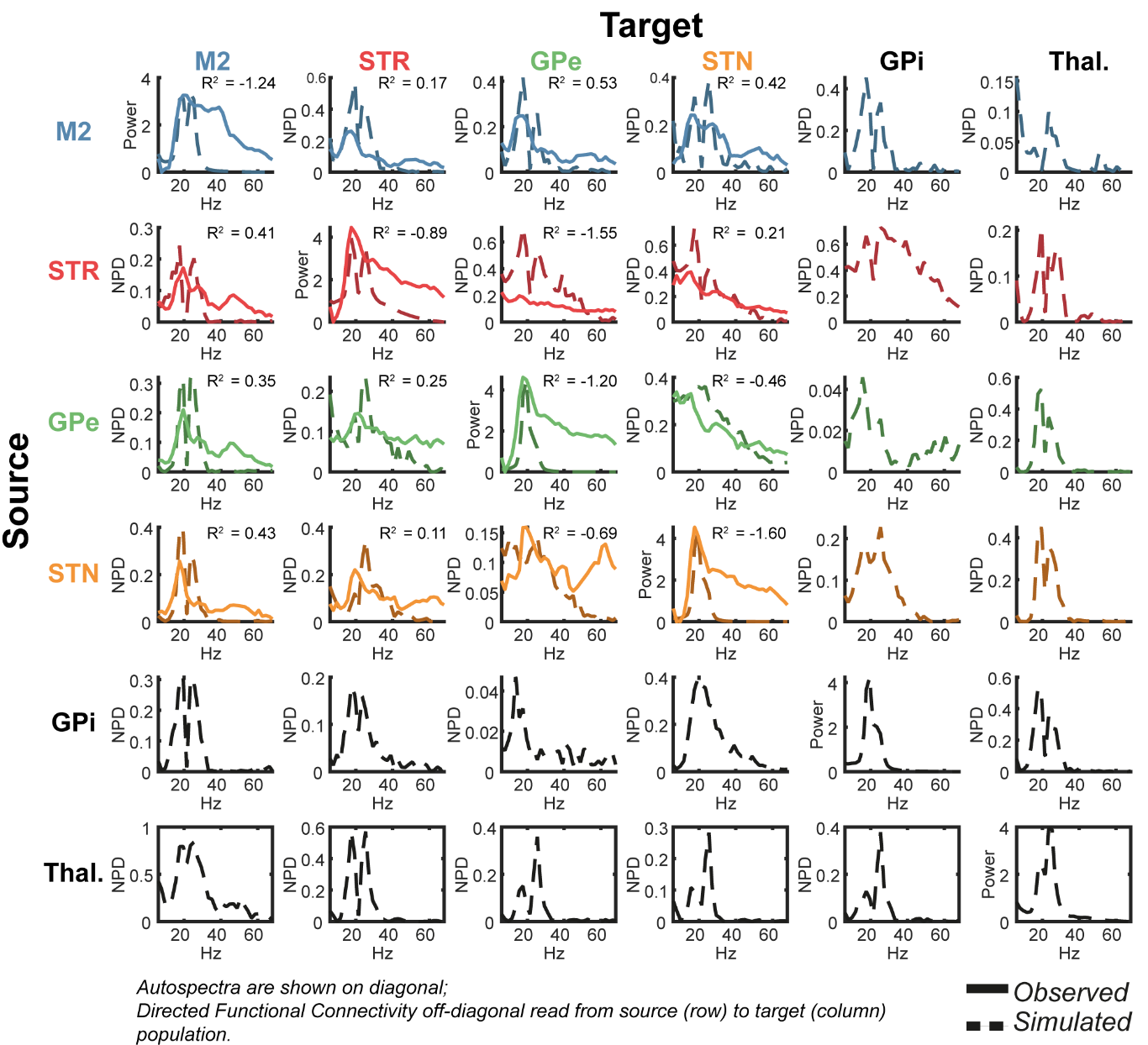


Fig S1 **–** **Full set of empirical data features: autospectra (diagonal) and directed functional connectivity (off-diagonal) used in the parameter optimization, and their resulting fits.** Empirical data (bold) is taken from a group level analysis of electrophysiological recordings made in the 6-OHDA rodent model of Parkinsonism (Mallet et al., 2008b, 2008a). ECoG was recorded from M2 region of cortex (blue), and LFPs from the STR (red), GPe (green), and STN (yellow). Data is shown prior to Gaussian smoothing that was applied before fitting. Predicted spectra are shown for the hidden nodes at GPi and Thalamus. Autospectra are placed along the diagonal. Directed functional connectivity (Non-parametric directionality; is on the off-diagonal and can be read from source (row) to target (column).
