# Supplementary material for: Stimulating at the right time to recover network states in a model of the cortico-basal ganglia-thalamic circuit
Source: PLoS Comput Biol. Author manuscript; Available in PMC 2022 Mar 29. (PMC8939795; doi:10.1371/journal.pcbi.1009887)
Supplement: S2 Appendix [file EMS143856-supplement-S2_Appendix.docx]

## S2 Appendix: Integration of Stochastic Differential Equations

The model incorporates a stochastic input $u_{i}$for each population which represents endogenous background activity. This input is given by:

$$u_{i}=C_{i}W,$$

*Equation (S2.1)*

where

$$W\sim N\left( 0,\sigma\right),$$

*Equation (S2.2)*

and $C_{i}$ represents a gain factor on the noise scaling the noise for population $i$. The noise $W$is drawn from a zero-mean normal distribution, with a standard deviation $\sigma$ that is set for the whole model. In the model presented here the stochastic innovations are independent of the state variable (i.e., they are additive) and a Euler-Maruyama (EM) scheme with a suitably small step size (*h* = 0.005 ms*;* less than half of the fastest time constant) is appropriate. This numerical scheme has been demonstrated to yield accurate results in similar models [1,2]. A formal assessment of the convergence of the EM scheme is beyond the remit of this paper but we refer the technical reader to [3,4]. For additive noise, this scheme follows on naturally from forward Euler and deploys a rescaling of the stochastic component by the square-root of the integration step $h$ to ensure fluctuations are obey a proper Weiner process (as per [5]):

$\hat{u_{i}}=u_{i}\surd h$.

*Equation (S2.3)*

To allow for settling of state equations, we set the initial states to be equal to zero, and then remove the initial transient (3s) as a burn-in.

## Supporting References

1. Ableidinger M, Buckwar E, Hinterleitner H. A Stochastic Version of the Jansen and Rit Neural Mass Model: Analysis and Numerics. J Math Neurosci. 2017;7: 8. doi:10.1186/s13408-017-0046-4

2. Palmigiano A, Geisel T, Wolf F, Battaglia D. Flexible information routing by transient synchrony. Nat Neurosci. 2017;20: 1014–1022. doi:10.1038/nn.4569

3. Buckwar E. Introduction to the numerical analysis of stochastic delay differential equations. J Comput Appl Math. 2000;125: 297–307. doi:10.1016/S0377-0427(00)00475-1

4. Baker CTH, Buckwar E. Numerical Analysis of Explicit One-Step Methods for Stochastic Delay Differential Equations. LMS J Comput Math. 2000;3: 315–335. doi:10.1112/s1461157000000322

5. Hansen JA, Penland C, Hansen JA, Penland C. Efficient Approximate Techniques for Integrating Stochastic Differential Equations. Mon Weather Rev. 2006;134: 3006–3014. doi:10.1175/MWR3192.1
