# Supplementary material for: Stimulating at the right time to recover network states in a model of the cortico-basal ganglia-thalamic circuit
Source: PLoS Comput Biol. Author manuscript; Available in PMC 2022 Mar 29. (PMC8939795; doi:10.1371/journal.pcbi.1009887)
Supplement: S2 Fig [file EMS143856-supplement-S2_Fig.docx]

## S2 Supplementary Figure – Analysis of multi-Gaussian fits to empirical data


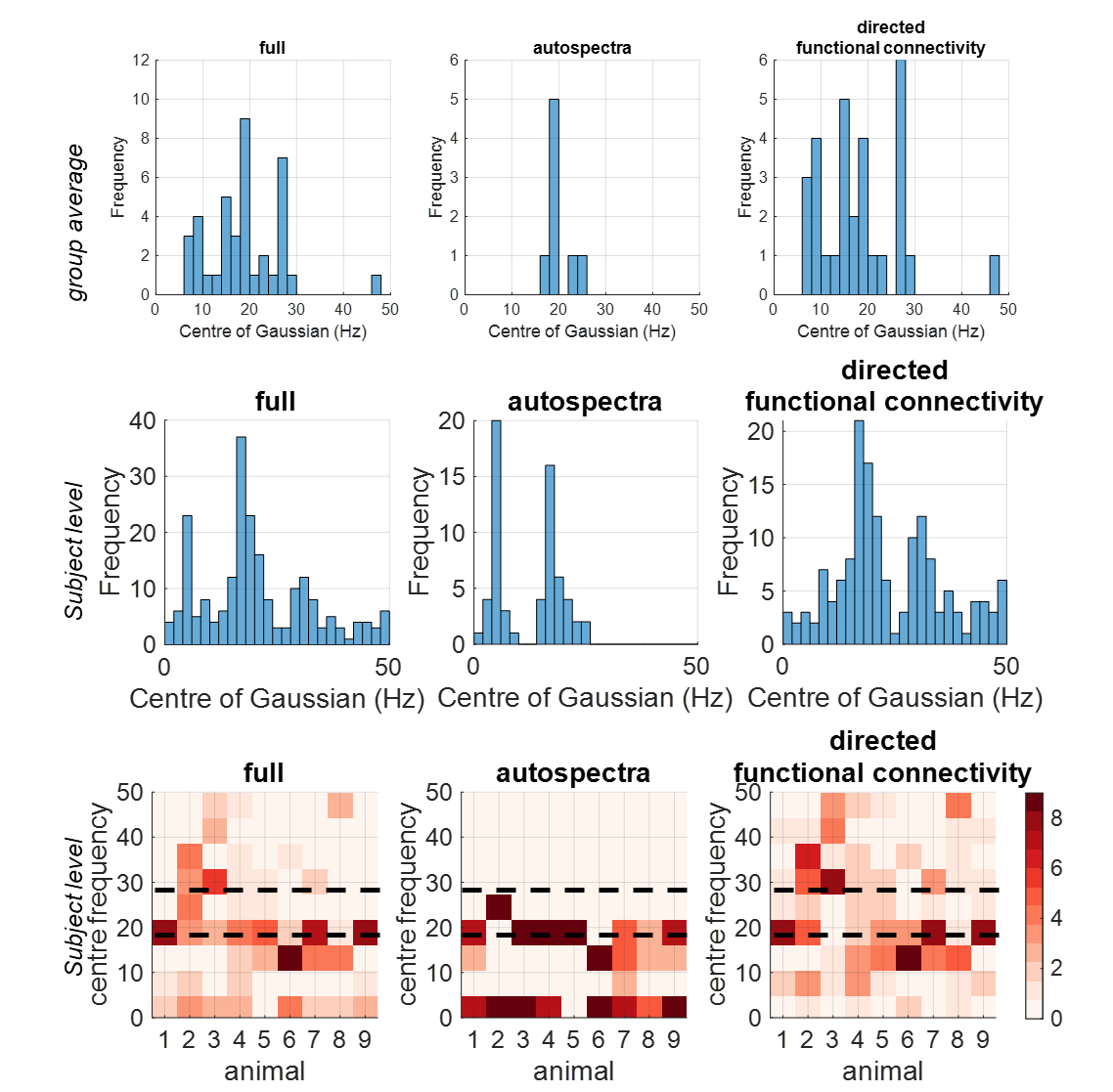


Fig S2 – **Histograms of centre frequencies for Gaussian distributions fit to spectral data features.** Coefficients representing individual means from sum of Gaussians falling between 0 and 50 Hz were examined across the data features. The histogram from the full data features shows 3 peaks at 5 Hz, 18 Hz, and 30 Hz. When considering the contributions from auto-spectra, peaks were predominantly centred around 18 Hz, whereas the directed functional connectivity exhibited both 18 Hz and 30 Hz peaks.
