# Supplementary material for: Stimulating at the right time to recover network states in a model of the cortico-basal ganglia-thalamic circuit
Source: PLoS Comput Biol. Author manuscript; Available in PMC 2022 Mar 29. (PMC8939795; doi:10.1371/journal.pcbi.1009887)
Supplement: S3 Appendix [file EMS143856-supplement-S3_Appendix.docx]

## S3 Appendix: Candidate Model Space and Model Selection

The model used in this paper was selected from a set of 13 models designed to look at the role of four pathways and their interactions: the cortico-striatal indirect; the cortico-subthalamic hyperdirect; thalamocortical relay; and the subthalamic-pallidal feedback. In total we tested 6 families of models, each subdivided into two submodels (see below), to yield 12 initial models.

1. + indirect.
2. + indirect/ + hyperdirect pathway.
3. + hyperdirect / - indirect.
4. + indirect / - hyperdirect/ + thalamocortical.
5. + indirect / + hyperdirect/ + thalamocortical.
6. - indirect / + hyperdirect/ + thalamocortical.

The second level of families (i.e., x.1-2) investigates whether the reciprocal network formed by the STN and GPe is required to explain observed patterns of connectivity in the data. A 13^th^ model including a connection from GPe to GPi amongst the full model was also tested following a reviewer’s comment.

To compare between ABC fitted models we used a previously described model comparison procedure [1]. Briefly, the ABC approximated posterior distribution over parameters $P(\theta|D_{0})$can be used to approximate the model evidence $P\left( M | D_{0} \right)$. This estimate is made by drawing $N$ times from the posterior and then computing an exceedance probability to estimate the marginal probability of the *j^th^* model $M$ given data $D_{0}$ (the approximate model evidence):

$$P\left( M^{j} | D_{0} \right)\cong\frac{\# \rho\left( \mu_{n},\mu_{0} \right)\geq\epsilon^{*}}{N}$$

*Equation (S3.1)*

where $\epsilon^{*}$ is a threshold on the model error $\rho$ that is suitably small to give an acceptable fit on the data. If $\epsilon^{*}$is held constant and the data is identical between models, then the exceedance probabilities may be compared to yield the model that gives the most accurate fit. In practice we set $\epsilon^{*}$ to be the median error of all sets of models. We found that a model incorporating both the hyperdirect and subthalamo-pallidal pathways was the best candidate in describing the patterns of neuronal activity in recordings made in Parkinsonian rats. This posterior model fit is used for the simulations in this paper which we refer to as the *fitted* model and its structure is presented in figure 1. Specifically, we use the maximum a posteriori (MAP) estimate (the mode of the marginal posterior distribution over parameters) to specify the fitted parameters that we use as a baseline in the simulations presented in this paper.

## Supporting References

1. Toni T, Welch D, Strelkowa N, Ipsen A, Stumpf MP. Approximate Bayesian computation scheme for parameter inference and model selection in dynamical systems. J R Soc Interface. 2009;6: 187–202. doi:10.1098/rsif.2008.0172

2. Toni T, Stumpf MPH. Simulation-based model selection for dynamical systems in systems and population biology. Bioinformatics. 2009;26: 104–110. doi:10.1093/bioinformatics/btp619
