# Supplementary material for: Stimulating at the right time to recover network states in a model of the cortico-basal ganglia-thalamic circuit
Source: PLoS Comput Biol. Author manuscript; Available in PMC 2022 Mar 29. (PMC8939795; doi:10.1371/journal.pcbi.1009887)
Supplement: S3 Fig [file EMS143856-supplement-S3_Fig.docx]

## S3 Supplementary Figure – Alternative stimulation policies


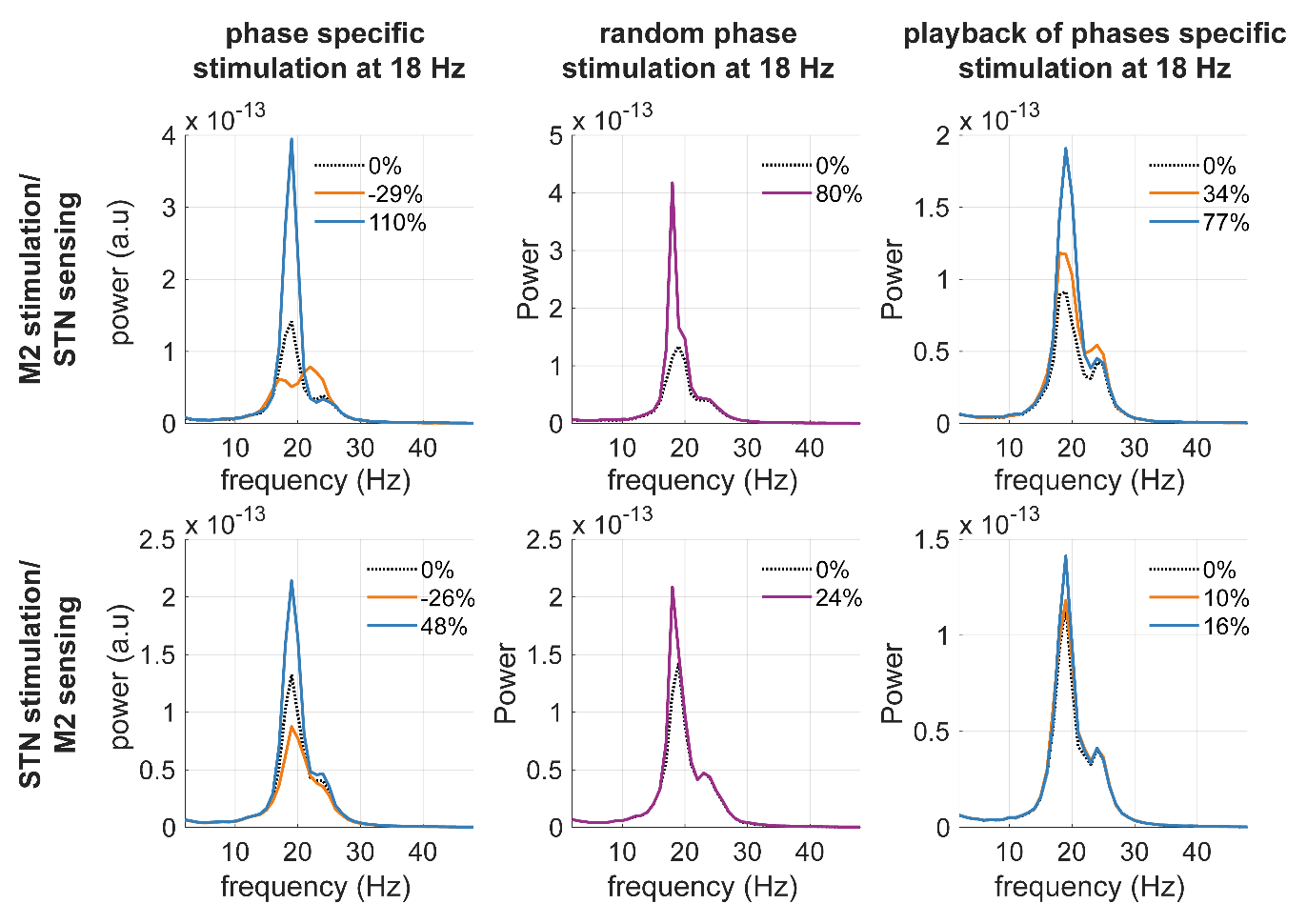
 Fig S3 – **Changes in the STN power spectrum from the dopamine-depleted model (black) during different stimulation modalities (coloured).** Stimulation was either delivered to M2 according to activity sensed at the STN (top row), or vice-versa (bottom row). Stimulation policies were either: 18 Hz phase locked (1^st^ column), 18 Hz random phase (2^nd^ column), or playback of 18 Hz phase locked stimulation (3^rd^ column). For phase specific stimulation, both the maximum amplifying (blue) and suppressive (orange) phases are shown, or in the case of non-phase specific, the singular stimulation effect is shown (purple). Stimulation was delivered when β_1_ band envelope exceeded the 75^th^ threshold during phase specific and random phase stimulation. Percentage changes in the β_1_ band power compared to baseline are indicated as inset text.
