# Supplementary material for: Stimulating at the right time to recover network states in a model of the cortico-basal ganglia-thalamic circuit
Source: PLoS Comput Biol. Author manuscript; Available in PMC 2022 Mar 29. (PMC8939795; doi:10.1371/journal.pcbi.1009887)
Supplement: S4 Appendix [file EMS143856-supplement-S4_Appendix.docx]

## S4 Appendix: Validation of the Zero-Crossing Procedure for On-Line Phase Estimation and Predicted Effects of Signal-to Noise Ratio

On-line phase estimates were constructed in order to set control parameters for our model of a closed-loop, phase locked stimulation paradigm. The procedure for estimation is detailed in the main methods but here we provide a validation. This validation uses the long-time Hilbert estimated phase as a benchmark by which to compare accuracy of the phase recovery using the phase estimation algorithm. The degree of similarity between the two estimates was computed using the phase locking value (PLV; [1]) with a PLV of 1 indicating a perfect recovery. We used two signals in the validation: (1) 80s of 18 Hz sinusoid with additive noise; and (2) an 80s simulation of the STN from the fitted model reported in this paper. We also examined two update frequencies (the period between each online phase estimation procedure): 5ms and 25ms. The results of this validation are given in the table below. Recovery for the noisy sinusoid signal was more accurate that the simulated signal as there is no fluctuation in the underlying sine’s amplitude, whereas in the model rhythmic activity waxes and wanes. This is supported by the finding that PLV estimates of the recovery were more accurate when assessing only the parts of the signal that were greater than the median amplitude.

|  | Complete | | >50% Amplitude | |
| --- | --- | --- | --- | --- |
| Update Period (ms) | *5* | *25* | *5* | *25* |
| *Noisy Sinusoid (PLV)* | 0.957 | 0.952 | 0.9542 | 0.9355 |
| *Simulated Network (PLV)* | 0.7314 | 0.6942 | 0.866 | 0.8347 |

Table - **Table of phase locking values (PLV) from validation of on-line phase estimation.** Values indicate the consistency (1 being perfect correlation) of the on-line phase estimation algorithm and that of the off-line Hilbert calculated phase.


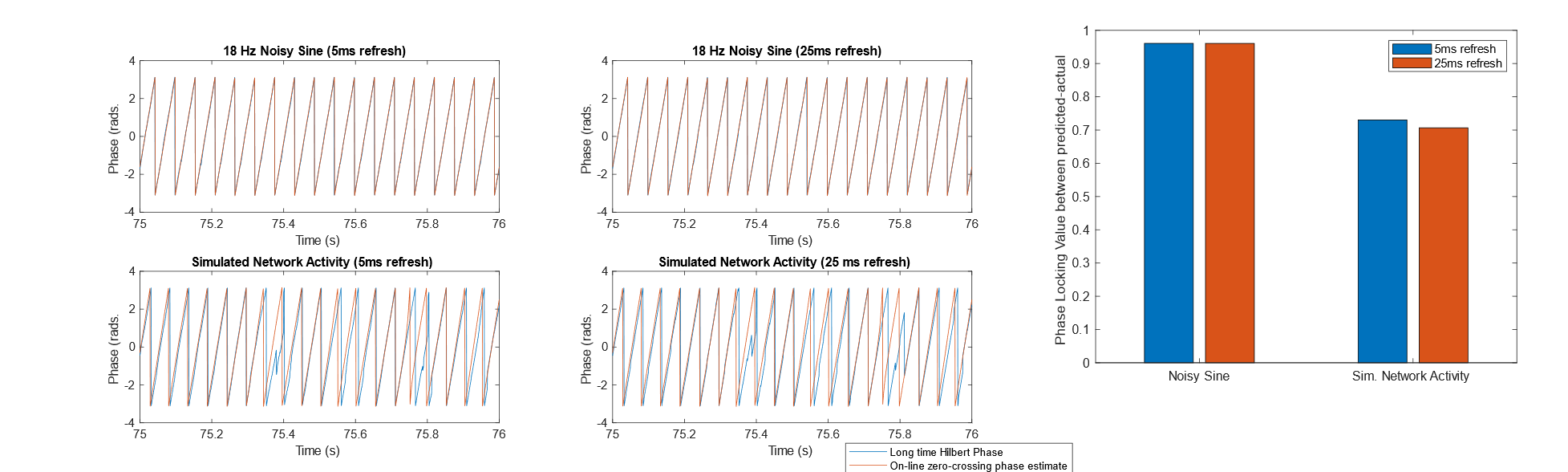


Figure – **Results from validation of on-line phase locking algorithm (zero-crossing detection and interpolation)**.

We also estimated the effect of varying signal-to-noise ratio (measured as SNR dB) upon the phase locking algorithm. We first used a noisy sinusoid signal (described above) and added Gaussian white noise as additive noise to modulate the SNR. Noise was given with a specific ratio with respect to the variance of the signal in order to achieve a range of observation scenarios from -20 dB to +10 dB. Again, we assess accuracy of the on-line phase estimate with respect to phase estimated from the Hilbert Transform, using the PLV.

The results shown in the figure below indicate that on-line phase estimates are stable in the range -5dB to +10 dB (i.e., good signal quality). Below -10dB, errors in phase estimation introduced by observation noise confound the ability of phase specific stimulation to achieve suppressive effects on beta rhythms. Further, at very low SNR (< -15 dB) phase locked stimulation has an entrainment effect causing exclusively amplifying effects on beta power.
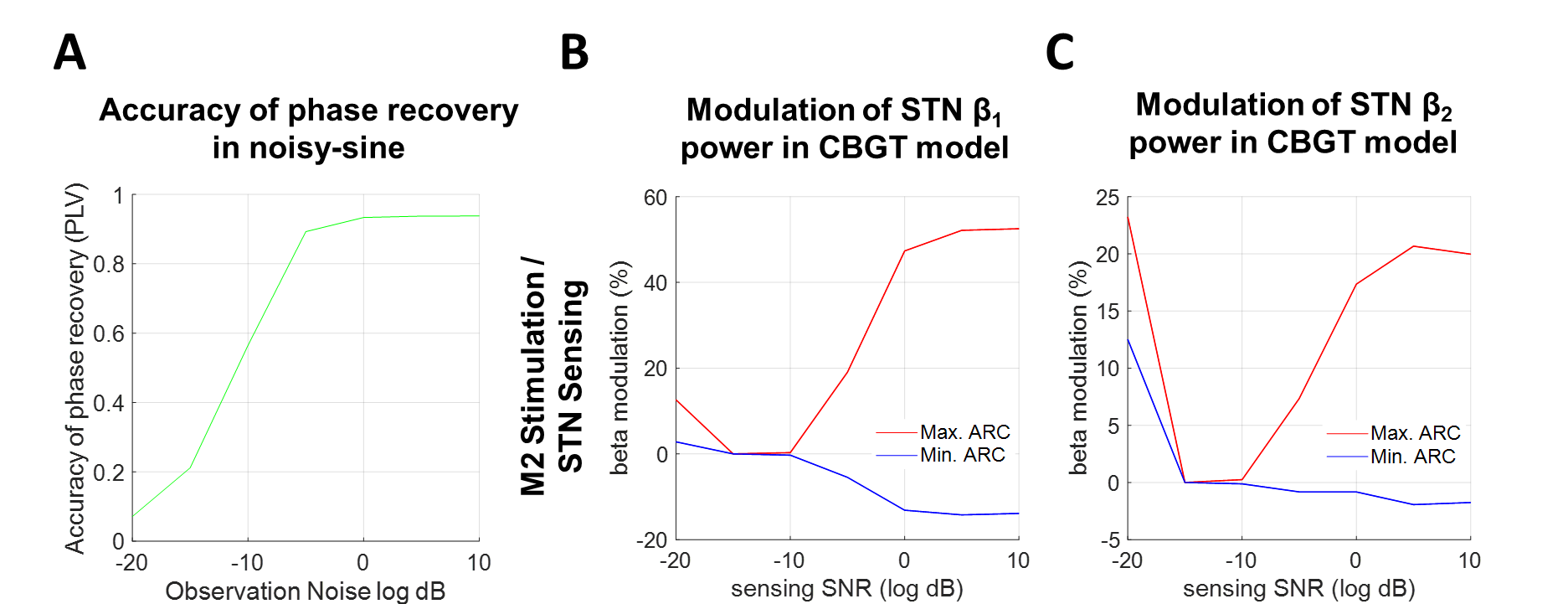


Figure – **Results from analysis of effect of signal SNR upon of on-line phase locking algorithm (zero-crossing detection and interpolation)**. **(A)** Effects of signal SNR on accuracy of phase estimate in noisy sine model, using the PLV to compare against the long-time Hilbert phase estimate. **(B and C)** Effects of signal SNR on the ability of phase specific stimulation to modulate STN beta rhythms in the CBGT model.

## Supporting References

1. Lachaux JP, Rodriguez E, Martinerie J, Varela FJ. Measuring phase synchrony in brain signals. Hum Brain Mapp. 1999;8: 194–208.
