# Supplementary material for: Stimulating at the right time to recover network states in a model of the cortico-basal ganglia-thalamic circuit
Source: PLoS Comput Biol. Author manuscript; Available in PMC 2022 Mar 29. (PMC8939795; doi:10.1371/journal.pcbi.1009887)
Supplement: S4 Fig [file EMS143856-supplement-S4_Fig.docx]

## S4 Supplementary Figure – Analysis of stimulation effects when using different control parameters


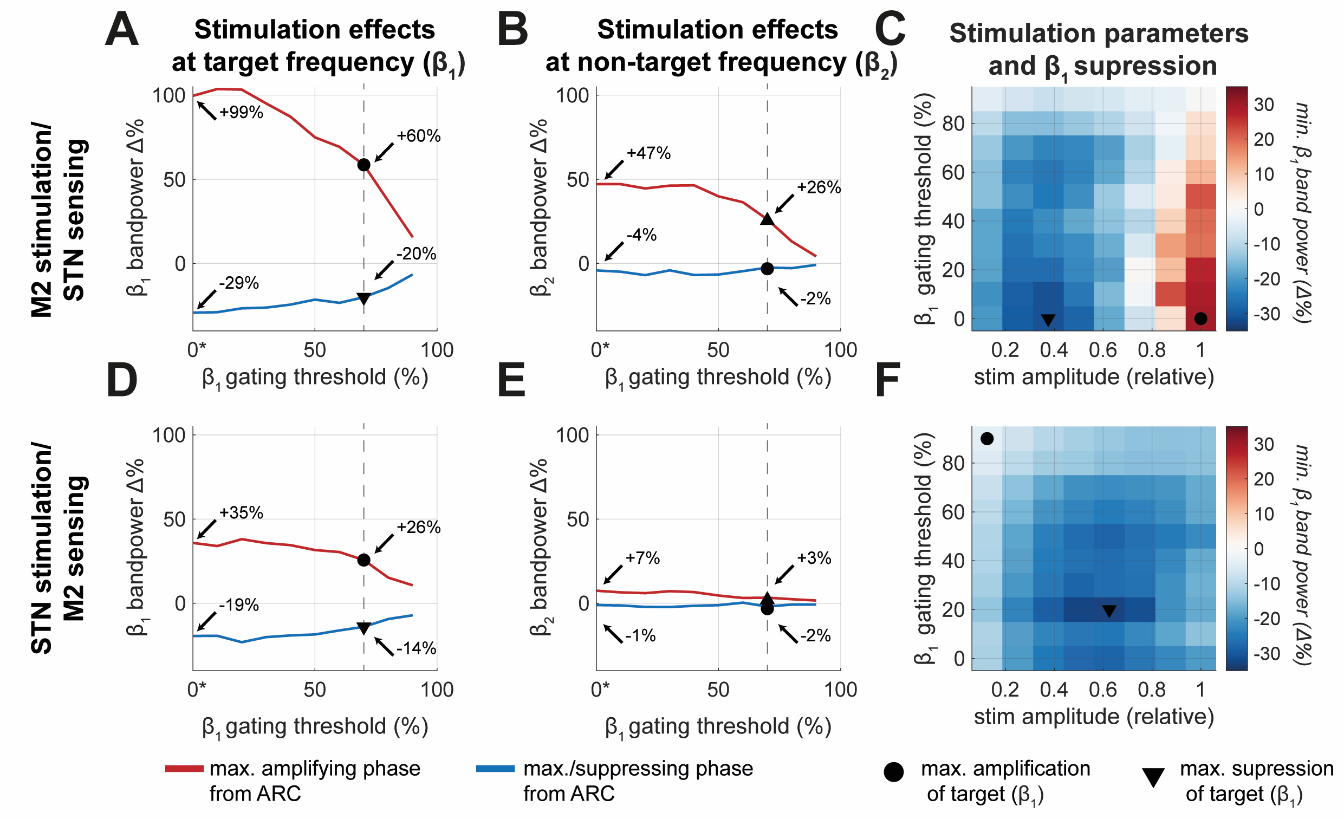
 S4 Fig S4 – **Analysis of stimulation effects upon β_1_/β_2_ power when using different parameters to control phase-locked M2 stimulation using activity sensed in the STN (1st row) or vice-versa (2^nd^ row).** **(A)** Effects of stimulation upon either the maximum (red) or minimum (blue) of the amplitude response curves (ARCs) for β_1_ power (the rhythm targeted by stimulation); or **(B)** β2 power (untargeted, auxiliary rhythm). The dashed lines indicate the 75^th^ percentile (used for stimulation control in the main text), with minimum (triangle) and maximum (circle) effects on the targeted β_1_ band indicated. **(C)** Heatmap of ARC minimum for β_1_ power when modulating both the gating threshold (percentile of envelope) and stimulation amplitude (fraction of variance of noise process in base model). The global max. suppression (triangle) and amplification (circle) of β_1_ power, are indicated. **(D, E, and F)** Same as A, B, and C but for a stimulation control policy using STN stimulation according to activity sensed in M2.
