# Supplementary material for: Stimulating at the right time to recover network states in a model of the cortico-basal ganglia-thalamic circuit
Source: PLoS Comput Biol. Author manuscript; Available in PMC 2022 Mar 29. (PMC8939795; doi:10.1371/journal.pcbi.1009887)
Supplement: S5 Fig [file EMS143856-supplement-S5_Fig.docx]

## S5 Supplementary Figure – Additional analyses of phase locked stimulation effects

##
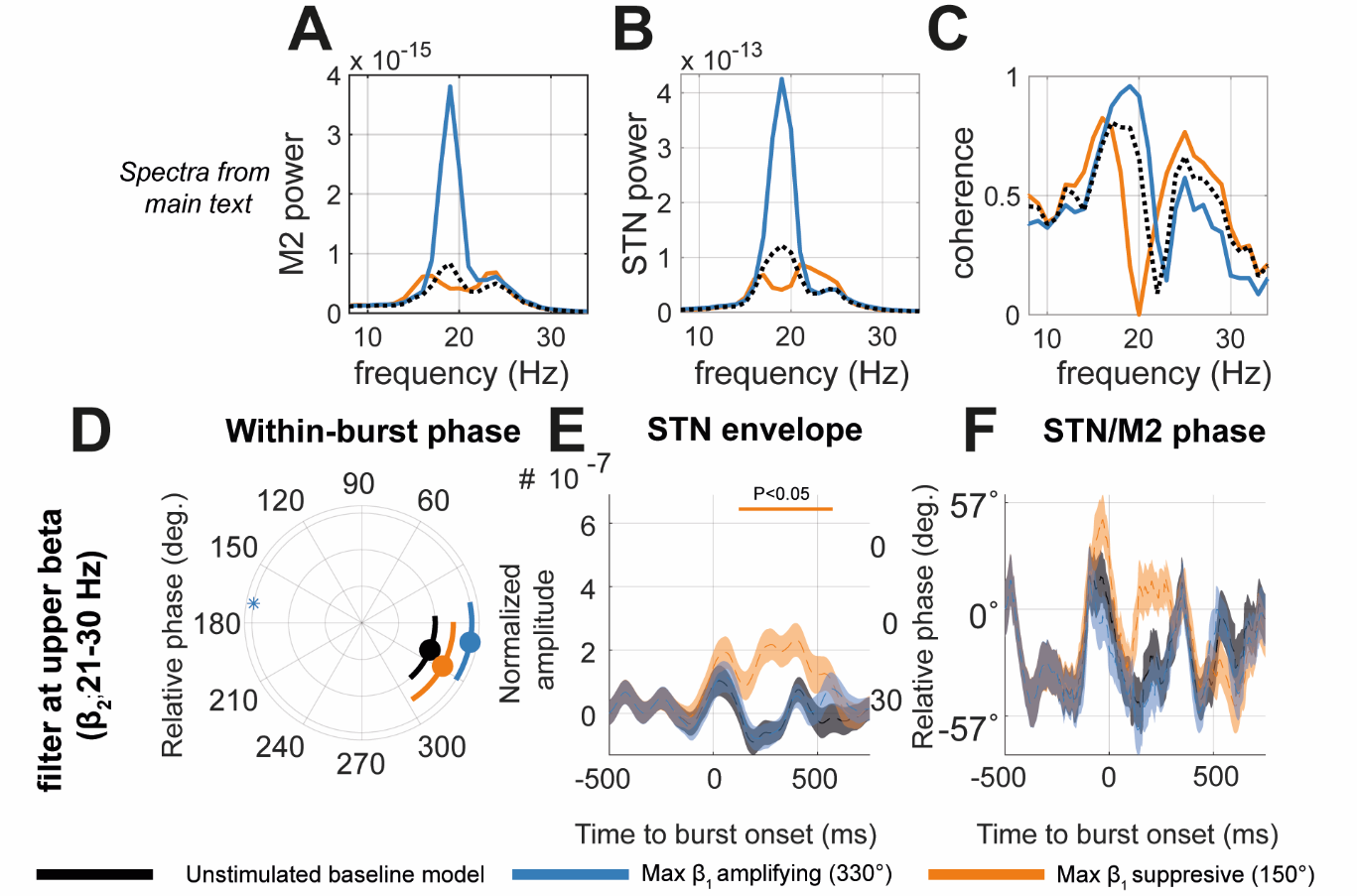


Fig S5 - **A model of dual-site controlled stimulation of the subthalamic nucleus using signals sensed in the motor cortex can modulate spectral patterns of beta frequency activity.** Spectra for maximal suppressive (orange) or amplifying (blue) stimulation phases compared with the unstimulated model (black), shown for **(A)** M2, **(B)** STN, and **(C)** STN/M2 coherence. **(D)** Radar plot of baseline changes in STN/M2 phase difference at non-targeted β_2_ frequencies. Circles indicate median, with bars giving the circular standard deviation. *indicate significant Rayleigh test for difference in phases from baseline model. **(E)** Analysis of the STN amplitude envelope filtered at β_2_ frequencies. Traces are mean+/- S.E.M timelocked to burst onset at t = 0. Bars indicate significant cluster-statistics (two-sample t-test, n=500, α < 0.05) for deviation from out-of-burst activity (see methods). Note emergence of peak of β_2_ burst is delayed by ~100ms. **(F)** Analysis of M2/STN β_2_ phase difference (centred relative to the mean phase at 0°).
