# Supplementary material for: Stimulating at the right time to recover network states in a model of the cortico-basal ganglia-thalamic circuit
Source: PLoS Comput Biol. Author manuscript; Available in PMC 2022 Mar 29. (PMC8939795; doi:10.1371/journal.pcbi.1009887)
Supplement: S6 Fig [file EMS143856-supplement-S6_Fig.docx]

##
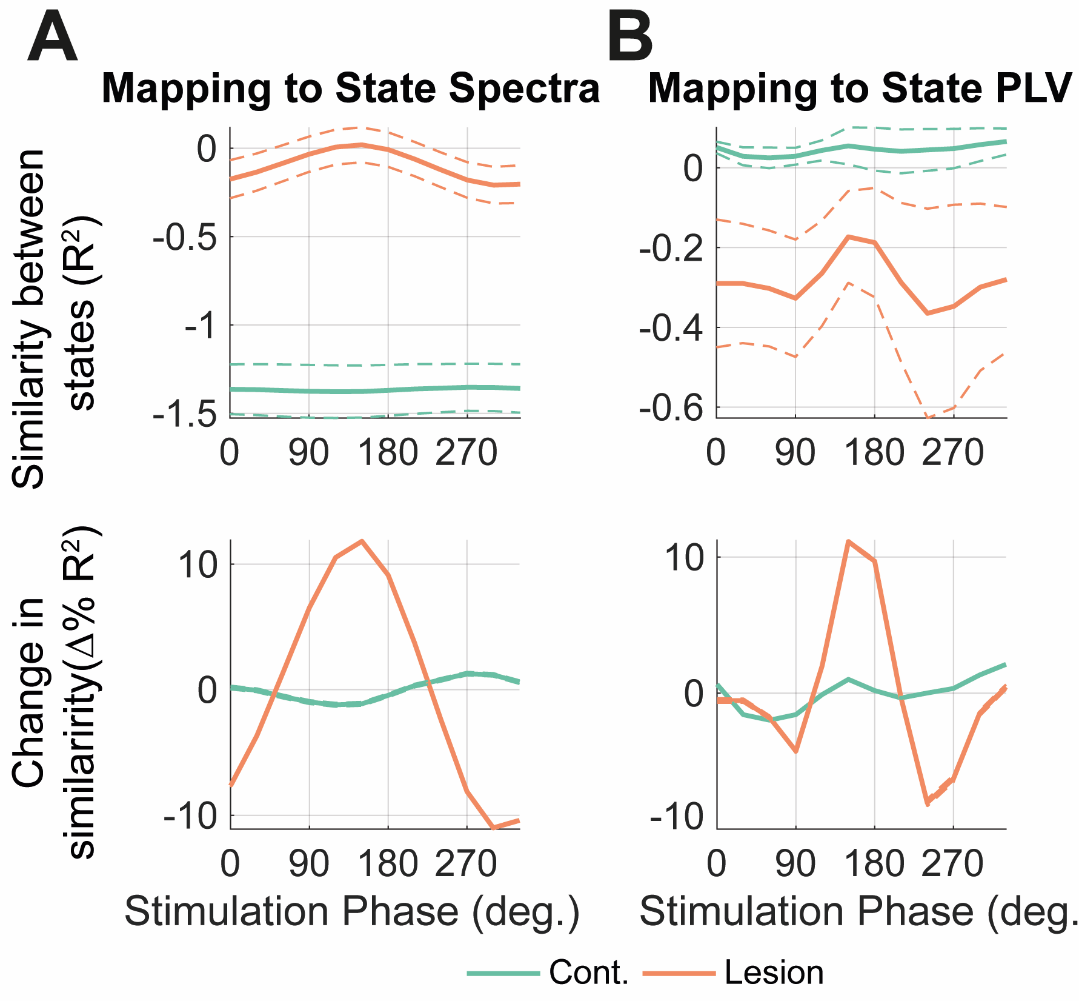
S6 Supplementary Figure – Stimulation recovery of empirically derived network state

Fig S6 – **Examination of the recovery of empirical network states by cortical stimulation phase locked to the STN.** Spectral fingerprints were constructed from experimental recordings of the basal-ganglia from control (teal) and 6-OHDA lesioned (peach) rats (see section Characterizing Network States with Spectral Fingerprints) and compared to those computed with stimulation. Pairs of phase locking values with cortical ECoG was not included due to the large differences in SNR of these recordings not reflected in the model. Due to variation in phase response, both control and lesion curves were realigned with respect to the phase invoking maximal recovery of the lesion state. **(A)** Recovery of control spectra was poor, due to inability of stimulation to completely suppress beta (max. suppression ~30%). Dashed lines indicate the S.E.M. of estimates. Spectral recovery exhibited an antiphase recovery of lesion vs control states. **(B)** Recovery of patterns of pairwise PLV was greater for control states, although a similar antiphase relationship was found between the two.
