# Supplementary material for: Stimulating at the right time to recover network states in a model of the cortico-basal ganglia-thalamic circuit
Source: PLoS Comput Biol. Author manuscript; Available in PMC 2022 Mar 29. (PMC8939795; doi:10.1371/journal.pcbi.1009887)
Supplement: S7 Fig [file EMS143856-supplement-S7_Fig.docx]

## S7 Supplementary Figure – State Recovery when Using STN stimulation Phase Locked to Motor Cortical Activity


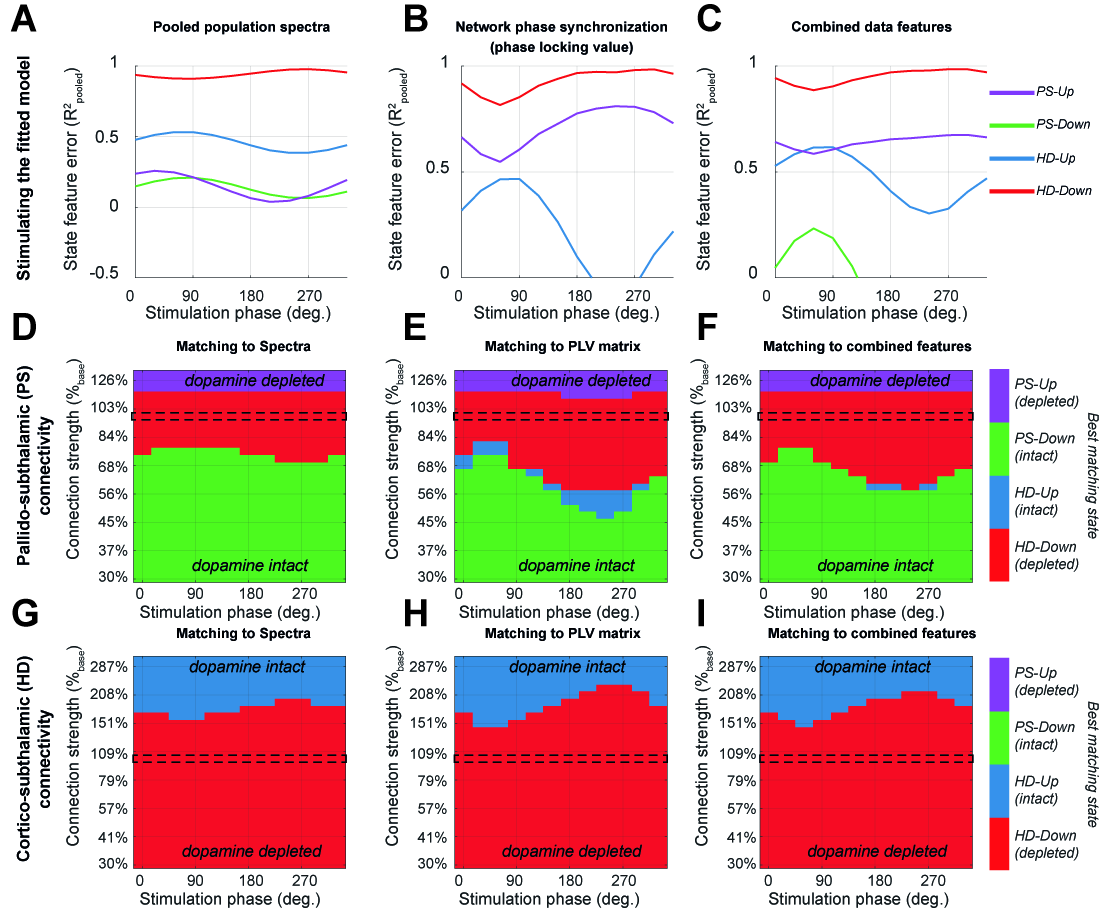


S12 Supplementary Figure – **Analysis of state recovery when using STN stimulation phase locked to activity sensed in the motor cortex.**  **(A, B, and C)** Stimulation was applied across 12 phases in the fitted model and data were compared (using pooled R^2^) to each network state using: the concatenated spectra (D); the matrix of PLV angle/magnitude (E); and the two features combined (F). These results show that spectral fingerprints from stimulation outcomes resembles that from different network states (i.e., a change in synaptic connectivity) depending upon the phase at which stimulation was delivered. **(D, E, F)** The above was repeated but when varying the connection strength of the PS pathway. Results are plot as a heatmap color-coded to indicate the best fitting state at each phase (x-axis; angle of stimulation relative to STN activity) and connection strength ( y-axis; percentage of fitted synaptic strength, dashed line indicates 100%- i.e., the model fit to the 6-OHDA lesion data and plot in D, E, and F). **(G, H, and I)** Same as D, E, and F, but for modulations of HD pathway strength.
