# Supplementary material for: Stimulating at the right time to recover network states in a model of the cortico-basal ganglia-thalamic circuit
Source: PLoS Comput Biol. 2022 Mar 4;18(3):e1009887. doi: 10.1371/journal.pcbi.1009887 (PMC8939795; doi:10.1371/journal.pcbi.1009887)
Supplement: Supplementary file 2 [file pcbi.1009887.s012.docx]

## S1 Table 

## Table of External Software

| Toolbox Name | Author | Year | Source/Reference |
| --- | --- | --- | --- |
| allcomb | ‘Jos’ | 2018 | [https://uk.mathworks.com/matlabcentral/fileexchange/10064-allcomb-varargin](https://uk.mathworks.com/matlabcentral/fileexchange/10064-allcomb-varargin) |
| boundedline-pkg | Kelly Kearney | 2015 | [ https://github.com/kakearney/boundedline-pkg](https://github.com/kakearney/boundedline-pkg) |
| brewermap | Stephen Cobeldick | 2014 | [ https://github.com/DrosteEffect/BrewerMap](https://github.com/DrosteEffect/BrewerMap) |
| Fieldtrip | Donders Institute, Radbound University | 2020 | [https://www.fieldtriptoolbox.org/](https://www.fieldtriptoolbox.org/) |
| linspecer | Jonathan C. Lansey | 2015 | [ https://github.com/davidkun/linspecer](https://github.com/davidkun/linspecer) |
| neurospec 2.2 | David Halliday | 2018 | [ https://www.neurospec.org/](http://www.neurospec.org/) |
| ParforProgMon | Dylan Muir, Willem-Jan de Goeij, The MathWorks, Inc. | 2016 | [ https://github.com/DylanMuir/ParforProgMon](https://github.com/DylanMuir/ParforProgMon) |
| splitvec | Bruno Luong | 2009 | [ https://uk.mathworks.com/matlabcentral/fileexchange/24255-splitvec](https://uk.mathworks.com/matlabcentral/fileexchange/24255-splitvec) |
| SPM 12 | Wellcome Centre for Human Neuroimaging, University College London | 2020 | [https://www.fil.ion.ucl.ac.uk/spm/](https://www.fil.ion.ucl.ac.uk/spm/) |
