# Supplementary material for: Stimulating at the right time to recover network states in a model of the cortico-basal ganglia-thalamic circuit
Source: PLoS Comput Biol. 2022 Mar 4;18(3):e1009887. doi: 10.1371/journal.pcbi.1009887 (PMC8939795; doi:10.1371/journal.pcbi.1009887)
Supplement: Supplementary file 3 [file pcbi.1009887.s013.docx]

PCOMPBIOL-D-21-01076

**Stimulating at the Right Time to Recover Network States in a Model of the Cortico-Basal Ganglia-Thalamic Circuit.**

*Timothy O. West, Peter J. Magill, Andrew Sharott, Vladimir Litvak, Simon F. Farmer, Hayriye Cagnan*

Dear editor,

We thank all of the reviewers for their extensive and insightful comments on the manuscript, based on which, we have made significant changes to our work. We have addressed all the reviewers’ concerns, and detail changes in the letter below. These changes are also highlighted as coloured text throughout the manuscript and supplementary information. 

*Note that, we identified a minor error in our analysis for figure 5 – namely that we were calculating linear error (R*^*2*^*) on phase angles which are a circular quantity.* 

*We now use the circular R*^*2*^ *and have updated both the figure and relevant methods section of the manuscript as follows:*

“In the case where we computed error of phase angles (figure 5 only), we estimated the circular R^2^, using the square of the mean resultant length [99]. When computing the pooled measure, we include the circular estimate within the sum of equation (8).”

L821-823

**Reviewer #1:**

1.1. “This manuscript describes the implementation and analysis of a model of the cortical-basal ganglia loop relevant to the contributions of abnormal oscillatory neural activity present in PD and suppression of this activity (and symptoms) by brain stimulation. The results are potentially interesting, but the initial paramterization of the model yields only a modest match the (quite) limited experimental data, the focus on beta “burst” activity is not well justified, especially in light of prior results of phase-specific stimulation in vivo (Sanabria et al), and the analysis of the therapeutic potential of phase-specific stimulation must be compared to other “control” forms of stimulation to put the effects in context.”

We thank the reviewer for raising these important issues, which we address in the following segments: (A) relationship of artificial network states to experimental data (c.f., 1.2, 1.4); (B) comparison to alternative, non-phase specific stimulation (c.f., 1.7, 1.8); and (C) the relevance of burst activity to stimulation (c.f., 1.9, 1.11, 1.13). Concerns regarding model fit (c.f., 1.15) were raised also by R3, and some of our responses are common to both (see also 3.4 and 3.5).

1.2. “**Major:** The analysis of the ability of phase specific stimulation to restore “healthy” brain states is unclear and the presentation of these results is somewhat convoluted.”

We thank the referee for raising this important point. We made a number of changes to the manuscript to highlight the physiological relevance of the hypothesised states (please see responses 1.3, 1.4, and 1.6).

To better understand how stimulation could restore “healthy” brain states, we now explore whether the modelled stimulation is able to recover fingerprints derived from the experimental recordings: control animals representing “healthy” brain and 6-OHDA lesion representing experimental Parkinsonism. The model can recover patterns of network synchrony (but not spectra) in dopamine intact, control animals, despite never being fit to this data. Importantly, recovery of empirical states from recordings of individual animals showed a clear antiphase relationship between control and lesion, mimicking that seen in the artificial states.

We add to the supplementary information:

“**S6 Supplementary Figure – Stimulation recovery of empirically derived network states**

S6- Supplementary Figure – **Examination of the recovery of empirical network states by cortical stimulation phase locked to the STN.** Spectral fingerprints were constructed from experimental recordings from control (teal) and 6-OHDA lesioned (peach) rats (see section Characterizing Network States with Spectral Fingerprints) and compared to those computed with stimulation. Due to variation in phase response, both control and lesion curves were realigned with respect to the phase invoking maximal recovery of the lesion state. **(A)** Recovery of control spectra was poor, due to inability of stimulation to completely suppress beta (max. suppression ~30%). Dashed lines indicate the S.E.M. of estimates. **(B)** Recovery of patterns of pairwise PLV was better (mean R^2^ = 0.34), and showed an antiphase recovery of lesion vs control states (average modulation of R^2^ = ±5%). 

SI L56-64

We refer to this new analysis in the results:

“In line with this, recovery of network states, linked to empirically derived control/lesion connectivity patterns (supplementary figure 6), exhibited an antiphase relationship and showed that given an optimal phase alignment, stimulation could recover patterns of phase synchronization seen in empirical dopamine intact network (mean R^2^ = 0.34). Note however, the ability of stimulation to recover spectral fingerprints derived from dopamine intact control animals (R^2^< -1) was poor. Spectral profiles derived from control animals did not contain a distinct peak in the beta band which gave rise to this poor performance. Complete suppression of beta rhythms is not reproducible with the 17% maximal β_1 _suppression achievable with modelled stimulation.”

L337-344

We add to the methods:

“Empirical states were constructed in the same way- computing the fingerprints of spectra and PLV individually for each of the nine animals. Note empirically derived spectra were corrected for 1/f background (as detailed in section *Data Features used for Model Estimation: Spectra and Directed Functional Connectivity*).”

L698-701

“Note that when comparing simulated with empirical states, we ignored features including GPi and Thal., as these were not available in the original data. Due to differences in the phase dependent recovery of empirical states, curves from both control and lesion conditions were realigned with respect to the phase attaining maximum recovery of the lesion state.”

L822-825

1.3. “Indeed, the assertion, ‘the ability to accurately recover states related to a deprecated HD pathway, suggests that M2 stimulation phase locked to STN may have the potential to restore network activity related to this pathway following degeneration related to dopamine depletion.’ is somewhat upside down, as presumanly one would want to use stimualtion to replicate the HDup state (and similarly upside down for the PSup state).”

We thank the reviewer for pointing this out. Indeed the above sentence was inaccurately phrased. We now correct it to indicate the aim is restore states relating to the upregulated HD pathway which we link to the healthy state:

“Furthermore, the ability to accurately recover states related to an upregulated HD pathway, suggests that M2 stimulation phase locked to β_1_ activity in the STN may have the potential to restore network activity following degeneration of this same pathway in response to dopamine depletion.”

L333-336

1.4. “Prior to an extensive analysis of a range of PS and HD connection strengths (many of which seem extreme), it would be much clearer to create a prototype of a PD state, with contributions from both PSup and HDdn…”. 

We have now revised the manuscript to emphasize the point that the fitted model corresponds to data recorded in the dopamine-depleted (i.e., 6-OHDA lesion) state, with all connections in the circuit contributing to this state. We chose to separately modulate the HD and PS connections in our artificial states, in order to isolate their influence and demonstrate pathway specific effects of phase specific stimulation. 

We now remind the reader throughout the text, that the “fitted” state pertains to the model that has had parameters constrained using the data from the 6-OHDA lesioned animals:

“the fitted model (i.e., the 6-OHDA lesioned state)…”

L169-170

“The fitted model (i.e., the 6-OHDA lesioned state…”

Fig. 2 legend

“…in the model fitted to the 6-OHDA lesioned state…”

L217

Regarding the range of PS and HD connection strengths , we now update the ranges to consider the existing literature:

“Previous experimental work highlighted a 70% increase in the amplitude of inhibitory post synaptic currents at the STN following dopamine depletion [11], equating to a *PS-Down* state with roughly 30% connectivity of the 6-OHDA lesioned model (i.e. fitted model). We set a *PS-Up* state with connectivity strength that elicited roughly a doubling of broadband β power in the STN (c.f. upper range of effective beta band modulation reported in [25]). Similarly, we set a *HD-Down* state also at 30% connectivity of the fitted model, and an *HD-Up* state yielding a doubling of broadband β power.”

L669-674

1.5. “…and examine effects of phase-specific stimulation therein, and specifically the ability to recover the fully connected state”

Please see our response to 1.2 which details a new analysis on the recovery of the empirical, “fully connected state”.

1.6. “It is not clear clear in the analyis of 5G-L what the match is to the fully connected (ie, healthy) network, and it is not therapeutically relevant to access all network states in the unhelathy network.”

To improve the readers ability to correspond artificial states to those hypothesized, we have modified figure 5G-L to indicate the relationship between each region and the healthy/pathological state.

1.7. “The assertion that, ‘the potential for precisely timed stimulation to compensate for weakening of corticosubthalamic transmission’ and ‘may be restored by well-timed stimulation’ require comparisons to phase independent stimulation, as continuous stimulation can rescue the PSup and HDdn cases, as observed exprimentally.”

This is an important point, and we now include several stimulation modalities including non-phase locked stimulation at 18 Hz, as well as playback of phase-locked stimulation at 18 Hz, neither of which could reach the levels of efficacy achievable with phase specific stimulation in this model.

We add to the methods:

“Alternative stimulation strategies were also tested. Specifically, we used phase non-specific stimulation at 18 Hz, using the above gating criteria but with fixed instantaneous frequency (i.e., constant phase). As a second control, designed to identify whether spectral effects resulted from the phase tracking algorithm, we also “played back” stimulation patterns [89] but in a different model instance, where the actual phase was no longer matched to the model dynamics.”

L806-810

We include a new section to supplementary materials regarding different control policies. Note we also investigate the difference in sense-stim setups using either STN or M2 stimulation (left and right column; see response to comment 2.3).

“**S3 Supplementary Figure – Alternative stimulation policies**

S3 Figure – **Changes in the STN power spectrum from the dopamine-depleted model (black) during different stimulation modalities (coloured).** Stimulation was either delivered to M2 according to activity sensed at the STN (top row), or vice-versa (bottom row). Stimulation policies were either: 18 Hz phase locked (1st column), 18 Hz random phase (2nd column), or playback of 18 Hz phase locked stimulation (3rd column). For phase specific stimulation, both the maximum amplifying (red) and suppressive (blue) phases are shown, or in the case of non-phase specific, the singular stimulation effect is shown (green). Stimulation was delivered when β_1_ band envelope exceeded the 75th threshold during phase specific and random phase stimulation. Percentage changes in the β_1_ band power compared to baseline are indicated as inset text.”

SI L19-28

Notably, we find that non-phase locked stimulation is not effective in suppressing beta rhythms in this model. We add to the results:

“We also tested a number of non-phase specific control strategies, including sinusoidal 18 Hz (with random phase), as well as playback of phase-locked stimulation (see methods section Modelling Stimulation of Motor Cortex Phase Locked). None of these alternative stimulation strategies were able to achieve a suppression of beta rhythms in the model (supplementary figure S4; see also discussion).”

L263-267

1.8. “Further, the effects of phase specific stimulation appear suprisingly modest. Although it is stated that, ‘Maximal suppression of β1 is close to that reported in experimental work’, this is clearly not the case, where in PD, the beta power can be dramatically suppressed by either levodopa or continuous DBS. And similary, stimulation herein has quite modest effects on beta amplitude within bursts (4D).”

We agree that the effects are modest and now show that stimulation efficacy reflects the nature of the adaptive control strategy – i.e., here stimulation is delivered following detection of high amplitude beta bursts. Since we use the 75th percentile threshold to detect these bursts – the majority of power has already occurred by the time stimulation is delivered (please also see our response in 1.9). 

To unpack this, we now include a supplementary set of results outlining the relationship between the burst threshold for gating stimulation versus the maximum achievable suppression/amplification of beta rhythms. Notably we find that lowering the threshold increases suppression of β_1_ (i.e., the target rhythm) but at the expense of amplifying an auxiliary (non-targeted) β_2 _rhythm:

“**S5 Supplementary Figure- Comparison of stimulation outcomes with different thresholds to gate delivery**

S5 Supporting Figure – **Analysis of stimulation effects upon β**_**1**_**/β**_**2**_ **power when using different parameters to control phase-locked M2 stimulation using activity sensed in the STN (1st row) or vice-versa (2****nd** **row).** **(A)** Effects of stimulation upon either the maximum (red) or minimum (blue) of the amplitude response curves (ARCs) for β_1_ power (the rhythm targeted by stimulation); or **(B)** β2 power (untargeted, auxiliary rhythm). The dashed lines indicate the 75th percentile (used for stimulation control in the main text), with minimum (triangle) and maximum (circle) effects on the targeted β_1 _band indicated. **(C)** Heatmap of ARC minimum for β_1_ power when modulating both the gating threshold (percentile of envelope) and stimulation amplitude (fraction of variance of noise process in base model). The global max. suppression (triangle) and amplification (circle), are indicated. **(D, E, and F)** Same as A, B, and C but for a stimulation control policy using STN stimulation according to activity sensed in M2.”

SI L30-41

Further, we compare to literature regarding phase locked or adaptive stimulation, as well as comparison to L-Dopa related suppression:

“We report here that stimulation of M2 could achieve a maximum of ~30% suppression of STN β_1 _rhythms, similar to the ~25% suppression achieved experimentally in a 6-OHDA rodent model [1]. Similar suppressive effects were also observed during adaptive DBS in humans (~14% beta power reduction in [2]) which was linked to a ~50% reduction in blinded hemi body UPDRS. It should be noted that approximately 60% suppression of beta power is observed following levodopa administration [3]. The range of amplification and suppression reported here is dependent upon both the threshold level chosen to gate stimulation delivery and stimulation amplitude (see supporting figure S5).”

L426-437

1.9. “The focus on and relevance of beta bursts in the present context is not clear, and this raises several imporant issues. First, there are substantial limitations to using a threshold to detect burst activity (see for example Anderson et al 2020 and Schmidt et al 2020) and average burst activity cannot be separated from average beta amplitude.”

We thank the referee for highlighting this important point. We chose to investigate beta bursts from a practical standpoint; phase estimation for time-locked stimulation requires periods of high SNR to accurately estimate phase dynamics. We now incorporate the following segment into the methods:

“We employ a burst definition based on the 75th percentile of the amplitude envelope. This is an important parameter which influences the efficacy of stimulation, as it sets a bound on the gating of stimulation. This choice is driven by the dependency between instantaneous phase and SNR [4,5]. Note that in practice, stimulation can be delivered at much lower thresholds (e.g., 15th percentile, see [6]) or with no threshold at all (see supplementary figure S5).”

L717-721

We thank the reviewer for highlighting the work of Schmidt et al., (2020), especially with respect to outlining the dangers of using a common threshold across conditions. In line with this work, we do not use a common threshold in our analyses.

“Recent work has highlighted the limitations of using a threshold to detect bursts, especially where a common threshold definition is applied across data [7,8]. Here, we avoid such a comparison, and compute thresholds specific to the given network states.”

L721-724

With respect to the functional significance and robustness of the current thresholding techniques we remain uncommitted to any particular approach. From the context of stimulation, the threshold remains a control parameter to be optimized for a given cost function (and refer the reviewer to the points made in response 1.8).

“Our model contains a number of important parameters, such as stimulation amplitude, frequency, shape etc., that are not explored here. These quantities form important targets for optimization, that can facilitate recovery of a range of network states. We used a high threshold for burst detection, and to gate stimulation. This resulted in a trade-off (supplementary figure S5) that allowed us to clearly illustrate phase dynamics, but in tandem limited the efficacy of the stimulation.”

L482-486

1.10. “Second, there are increases in bursts for HDup as well as HDdn, and the increase in beta amplitude at the “onset of burst” (3D and 3I) is a circular argument as this is how the bursts were defined to begin with.”

We thank the referee for pointing this out and now remove these features from the results.

1.11. “Third, the exclusive focus is on phase alignment within bursts, but also imperative to consider phase alignment otherwise…”

We focus on phase alignment during bursts (i.e., periods of high amplitude β_1 _rhythms) since these are the periods of activity that we target with stimulation, and are hypothesized to yield symptom reduction *in vivo.* 

We add clarifying statements to the relevant sections of results:

“…we investigated the phase synchronization occurring within bursts of rhythmic activity since these are significantly altered in Parkinsonism [16,41,42], and are commonly targeted with closed-loop stimulation (section A Model of Dual-Site, Phase Locked Stimulation...).”

L185-187

And:

“We specifically looked at transient dynamics in phase corresponding to β_1_ bursts, as these periods of activity are later targeted with phase specific stimulation (section A Model of Dual-Site, Phase Locked Stimulation...).”

L220-222

We append the figure below which shows the changes in phase alignment (phase locking values) across the network when either computed “within-burst” or with length matched data randomly sampled across the simulated data. We note two things: (A) the pattern of phase locking across the network is largely preserved when disregarding bursts; but (B) there is a loss of spatial selectivity of network synchronization.

And add the following to the methods:

“Note that spectral fingerprints were computed “within-burst” (see section Definition of Transient Burst Events and Statistics) since (A) we aimed to characterize network properties that were directly attributable to high SNR in the beta range. Preliminary analyses indicated that periods of high SNR increase the spatial selectivity of network synchronization. (B) We later compare the fingerprints derived from spontaneous activity and those induced by stimulation. As we gate stimulation according to beta bursts, this ensured that analyses were directly comparable between the two conditions.”

L702-707

1.12. “… and the within burst phase in Hdup is not considered (and does not fit with broad assertions).”

We thank the reviewer for bringing this to our attention, we now discuss phase dynamics within the HD-Up state:

“However, the phase evolution in the states associated with a reduction in Parkinsonian symptoms and weaker β_1 _burst amplitudes (*PS-Down*- green trace, or *HD-Up* – blue trace) exhibited: (a) a larger, transient, deviation in phase, and (b) noisier dynamics that settled more slowly over the duration of the burst after the burst amplitude started reducing.”

L233-237

1.13. “Fourth, and with respect to the importance of bursts vis a vis phase specific stimulation, this is overstated in the Introduction, as Sanabria et al observed similar results using phase-locked stimulation in vivo without reliance on burst activity.”

As highlighted in 1.8 and 1.9, we agree that the significance of burst detection in relationship to stimulation effects, beyond providing segments of data with high SNR, is not established (nor a central aim) of this paper. We have now adjusted our introduction to reflect this. Note that in the new supplementary figure S4, we apply phase locked stimulation without a threshold which achieves better beta suppression.

We note that the experiments of Sanabria and colleagues did employ a threshold to control stimulation timing (15th or 25th percentile of the amplitude envelope). Given our analysis detailed in 1.8, we add to our discussion:

“Our analysis of using different amplitude thresholds for gating stimulation (supplementary figure S4) suggests that frequency specificity is reduced at lower thresholds (such as those used in [26]). This may accentuate effects on non-targeted frequency bands (i.e. amplification of β_2_). These findings emphasize the importance of optimizing the gating threshold.”

L449-453

1.14. “Further, as Sanabria et al have demonstrated the utility of phase-locked stimulation, the reader should be reminded that this is not the first demonstration of phase-locked stimulation reducing beta amplitude in the Discussion.”

We thank the referee for highlighting this important work and now reword the introduction:

“In Parkinsonism specifically, stimulation of the subthalamic nucleus (STN) has been shown to both amplify or suppress pathological beta oscillations, dependent on the specific phase targeted in both primate disease models [26] and patients [25].”

L89-91

and discussion:

“This modulation of an auxiliary, non-targeted rhythm at β2 is consistent with experimental results reported by Sanabria and colleagues, in which phase locked STN stimulation led to higher frequency beta activity (>18 Hz) in tandem with targeted suppression of rhythms at 11-17 Hz [25].”

L445-447

1.15. “The model appears to generate a narrow beta band oscillation, but overall a somewhat marginal match between the experimental spectra an the model spectra, even after extesnive avergaging and smoothing of the former.”

We now include the goodness-of-fits (R^2^) of the individual data features in supplementary figure S1. The model was fit using the mean-squared error which had the effect of biasing fits towards accurately fitting directed functional connectivity, at the expense of fits to the spectrum due to the large difference in scales between these two features. In a separate model (using a pooled R^2^ as the error function) in which fits were better for spectra (but did not well recapitulate functional connectivity), we show that the conclusions of the paper hold – please see our response 3.5, as well as 1.26.

Furthermore, we reinforce the relevance of bimodal features by providing a supplementary analysis on the Gaussian fits, used to smooth the experimental data. Again, the bimodality was most apparent in the directed functional connectivity (for which the original model was biased to fit) and is apparent across the majority of animals used in the study.

“S2 **Supplementary Figure – Analysis of multi-Gaussian fits to empirical data**

S2 Supplementary Figure – **Histograms of centre frequencies for Gaussian distributions fit to spectral data features.** Coefficients representing individual means from sum of Gaussians falling between 0 and 50 Hz were examined across the data features. The histogram from the full data features shows 3 peaks at 5 Hz, 18 Hz, and 30 Hz. When considering the contributions from auto-spectra, peaks were predominantly centred around 18 Hz, whereas the directed functional connectivity exhibited both 18 Hz and 30 Hz peaks.”

SI L11-17

1.16. “(Note, the line thickness and large spaces between dashes in the dashed lines makes it very difficult to compare the spectra in S1).”

We have now adjusted S1 to allow a better comparison between the model and data.

1.17. “Several important limitations are not addressed in the Discussion. First, the experimental data used to compare the model were collected under a complex cocktail of anesthetics and this can influence brain oscillatory activity.”

To address limitations arising from the experimental choice of anaesthetics we add:

“Furthermore, models were fit to activity recorded under urethane anaesthesia and may be only qualitatively similar to that seen in the awake animal. This may overemphasize pathways such as thalamocortical feedbacks that exhibit increased strength under anaesthesia [66]. However, urethane’s effects on neurotransmitter function are small compared to other anaesthetics [67], and dose dependency allows access to cortically activated states which most resembles the waking brain [35].”

L509-514

1.18. “Second, the mtor cortical stimulation is delivered as an intracellular sine wave, and this has substantially different effects than either epicortical or intracortical (extracellular) stimulation.”

We agree, and now add to our discussion limitations of this model in describing mechanisms underlying conventional high frequency DBS, as well as the spatial aspects of stimulation:

“The simplification of dynamics to a single lumped time constant per population can obfuscate the interaction between inputs on different time-scales. More realistic models of brain stimulation, such as high frequency (120 Hz) pulse trains for DBS, would require conductance models, as well volumetric models considering the spatial specificity of a given stimulation modality.”

L499-503

1.19. “**Minor:** It is not readily apparent that cDBS targets factor B. The authors suggest reduction in beta oscillations is sufficient for this claim, but the same claim could be made for levodopa.”

We now clarify this segment:

“DBS and Levodopa both achieve similar therapeutic effects, but ostensibly in different ways: stimulation modulates neural activity directly (particularly on the shorter time scale); whilst dopamine replacement causes changes in activity via the modulation of synaptic transmission… Recent work suggests by refining the pattern of stimulation to target bursts of high amplitude beta activity [22,24] or altering its timing to match specific phases of the oscillation [25,26], it is possible to improve the specificity of therapy”

L80-86

1.20. “Not sure that (presumably multi-synaptic) functional connectivities’ "spectral fingerprints" should be equated to known pathways such as the hyperdirect pathway (monosynapic connections).”

We agree that this is an important consideration, and we now stress how seemingly small changes in structure (e.g., the strength of a monosynaptic connection) can have non-trivial effects upon the wider functional connectivity of the network. Thus we relate each of the spectral fingerprints to a certain network-state, determined by the up- or down-regulation of the synaptic input to the STN.

 We add:

“These results highlight how singular changes to monosynaptic inputs to the STN can shape activity of the wider network, not just by altering the amplitude and frequency of oscillatory activity, but also by dictating the synchronization of different elements of the wider circuit. “

L401-403

1.21. “The radial dimension in Figs 3C&H 4C is not clear. In Fig 4, suppressive stimulation appears to decrease every reported metric except this (unlabeled) one. At different radial lengths, the same SD will appear to take up more space, so variability on this axis should have some justification.”

The radial dimension of these polar plots has no meaning, bars were offset (by a small amount) to reduce the effect the reviewer correctly points out.

We now clarify this in the legend for figure 3:

“Note that the radial dimension has no meaning, bars are offset for presentation purposes. Angular lengths should be interpreted with relation to the grid-lines.”

Fig 3. legend

And in figure 4:

“Note that the radial dimension has no meaning, bars are offset for presentation purposes. Angular lengths should be interpreted with relation to the grid-lines.”

Fig 4. legend

1.22. “Many times the authors refer to "transient phase synchronization" but show something a little bit different, the phase synchronization during transient amplitude events. True analysis of "transient phase synchronization" would be agnostic to amplitude values.”

We agree with the reviewer and adjust our wording accordingly:

“Examination of the Phase Reorganization of Population Activities during Transient Beta Bursts”

L212-213

“…estimate changes in the dynamics of phase synchronization during beta bursts.”

L219-220

“…transiently phase synchronized networks emerge during bursts of high amplitude beta activity ...”

L214-215

1.23. “The changes in phase near the onset of the bursts (Fig 3E,J) are difficult to interpret, as there are opposite changes in realtive phase across pro- vs anti-PD changes in connection strength, as well as substantial changes in phase at times other than near burst onset.”

We note this inconsistent change in phase in the results:

“Note that the direction of change in relative phase was not consistent across different states.”

L236-237

1.24. “The correlations of phase stability and beta amplitude (3F,K) are just that – correlations, and thee obserations, contradicted inone of four cases, are not supportive of the statement of causation, “bursts persist for the duration of a stable period of phase locking between STN and M2”.

We correct this statement in line with the reviewer’s comment.

“This supports the idea that maintenance of high amplitude activity accompanies periods of stable phase locking between STN and M2, although it does not determine whether these changes in phase drive those in amplitude or vice-versa (see A Model of Dual-Site, Phase Locked Stimulation... for a direct manipulation of phase in the model).”

L242-245

1.25. “The tenor of the statement, “high amplitude beta oscillations that are known to be electrophysiological correlates of symptom severity”, is too strong as there are myriad exceptions to this relationship, and it is most strong only for bradykinesia.”

We alter this statement accordingly:

“We frame our results in terms of Parkinsonian high amplitude beta oscillations that are known to be electrophysiological correlates of bradykinesia [14,35].”

L119-120

1.26. “The error that was used to compare iteratively versions of the model to the experimenal data during the fitting process is not clearly described and more specific information beyond, ‘the distance between the simulated and empirical data’, is necessary.”

We clarify:

“Explicitly, experimental data and simulations are compared by first transforming the two datasets using a common summary statistic and then computing the goodness-of-fit, in terms of the mean squared error across the features…” 

L638-640

1.27. “The motor cortex is asserted as an ideal stimulaton target, yet clinical stimulation of motor cortex in PD has led overall to highly vaiable and mostly disappointing results.”

We agree that a survey of past literature pertaining to therapeutic, cortical stimulation for PD is disappointing at best. We discuss this further:

“Despite this, experimental evidence for therapeutic effects of cortical stimulation are inconsistent (see review [60]), yet no closed-loop approaches as presented here have been tested. The dual effects of phase specific stimulation (i.e., achieving both amplification and suppression), combined with recent evidence that cortical stimulation can suppress pathological activity in vivo [50,61] highlight the clinical potential for well-timed cortical stimulation. Nonetheless, our modelling suggested that although STN stimulation paired to cortical sensing exhibited inferior state recovery (supplementary figure S12), it can can suppress STN β_1 _(figure S4). This setup could be readily implemented with current technology leveraging paired ECoG recordings with DBS [32,34].”

L474-481

1.28. “The assertion, “this agrees with studies implicating increased coupling of the STN/GPe loop in the emergence of excess beta synchrony in the CBGT circuit” should consider other (competing) hypotheses on the origins of increased bet synchrony including striatial and cortical sites.”

We now relate our findings specifically to the model of Pavlides et al. in which cortical beta and the role of thalamocortical feedbacks were considered:

“Weakening of the HD pathway may give rise to STN/GPe resonance at β1 frequencies, as resonant activity is undisrupted by mismatched β2 inputs arriving from the cortex. In agreement with other models exploring thalamocortical feedback [13], we suggest that β1 rhythms amplified by the STN/GPe loop can return to motor cortex- effectively reinforcing synchrony across the CBGT at this frequency. This potentially explains how reduced HD transmission in PD can be observed alongside elevated cortico-subthalamic coherence [9]. How this translates to of enhanced an STN firing rate seen in primate models [51] is unclear, and would require models incorporating both oscillatory dynamics and detailed synaptic conductances to explain. Our model is distinct from a previous dynamic causal modelling study in which effective connectivity of the HD pathway was found to be raised in the 6-OHDA state [49], a finding in disagreement with findings of impaired cortico-subthalamic efficacy [8,9], and likely arising due to the relative insignificance of the STN/GPe circuit in that model.”

L408-419

And with regards to models of pallido-striatal generators of beta, we add:

“…, inclusion of arkypallidal projections would also allow us to predict how alternative models considering pallido-striatal origins of beta oscillations [69] may alter response to stimulation.”

L514-523

1.29. “Not clear that R2 is an adequate metric to assess similarity of concatenated spectra, as values are largely bimodal.”

We assessed the performance of two alternative error functions, including the mean squared error, as well as root mean squared error and computed the same analysis (see figure below). We find there to be no major qualitative differences between our findings:

Please however see our opening comment in which we detail changes in the error function for phase angles to account for the circularity of this variable.

1.30. “Fig 4B insets too small to be legible.”

We remove and add to supporting figure:

“Corresponding spectra are shown in supporting figure S6.”

Fig 4. legend

1.31. “Figure 4 legend title inverted stimulation and recording locations and is incorrect.”

We correct the figure title accordingly

“A model of dual-site controlled motor cortical stimulation phase locked to signals sensed in the subthalamic nucleus, can modulate beta activity across the network”

Fig. 4 legend

1.32. “’Data’ is plural and verbs should be congugated as such.

We have corrected to “data were…” in all cases where appropriate.

1.33. “Line 552 “that are themselves be made up of”, delete ‘be’”

Corrected.

1.34. “Line 427 “Our results show that the expression STN beta” needs an “of””

Corrected.

1.35. “Avoid jargon “spectral fingerprints”, “phase slip”, ‘open a channel for synchronization", …”

We alter “phase slips” to “phase reorganization” and remove ambiguous phrases or unnecessary jargon.

However, we maintain the usage of “spectral fingerprints” following its meaning broadly defined in [33] We improve our definition in the introduction:

“We summarise the changes in neural population activity (i.e., change to spectra and functional connectivity) associated with different network states (i.e., changes to structural connectivity) using statistical summaries that we term *spectral fingerprints* [18].” 

L98-101

Furthermore, we reduce the usage of this term, and restrict it predominantly to discussion of the comparison between states, in which it is useful to abbreviate reference to a statistical summary capturing both phase synchronization and spectral shape.

2. **Reviewer #2:**

2.1. “Thank you for inviting me to review this manuscript. My main expertise is human and clinical neurophysiology, so my review mainly focuses on physiological plausibility of the findings and the clinical outlook. Overall, this is a very valuable and innovative approach to computational network models of the basal ganglia. It innovates previous approach in multiple domains, as it reliably produces oscillatory population activity and connectivity, allows characterization of temporal dynamics that have recently been the focus of DBS neurophysiology research and investigates the effect of phase specific closed-loop stimulation that is currently on the rise for neurotherapeutics. The main finding that phase specific stimulation has the potential to compensate for pathological network disruption is impactful.”

In response to the reviewer’s comments, we have made several key changes: we (1) performed a comparison of beta suppression and state recovery when using either cortical stimulation phase locked to STN activity, or vice-versa, in order to better motivate our choices in the main paper (c.f., 2.5, 2.7);  (2) improved the clarity of our methods and results (c.f., 2.3, 2.5); and (3) expanded our discussion to put forward plausible ways for testing our hypotheses (c.f., 2.4). We also now include a comparison between different sensing/stimulation pairs and expand our analysis on the impact of different stimulation parameters.

2.2. “**I have few comments that should be addressed:**

1. One of the major results of the model dynamics is the definition of a shift from a healthy state associated with hyperdirect pathway driven activity with low pallidosubthalamic connection strengths to a severely dopamine depleted state with lower hyperdirect drive and increased coupling in the pallidosubthalamic pathway. Similar patterns have indeed been reported in experimental animal studies (Tachibana 2011). However, the latter and other studies have also demonstrated that firing rates in the parkinsonian state in the STN are significantly increased. How can the authors explain this reproducibly demonstrated increase in firing of the STN if the major glutamatergic input is decreased and the pallidosubthalamic inhibitory input is increased? Can this be contextualized to the effect of dopamine on pathway specific striatal spiny neurons?”

This is a very important point, and has formed much of the discussion introduced by previous authors such as Chu et al., (2015) as to how decreased excitatory drive to the STN can indeed drive increased firing rates, as well as cortico-subthalamic coherence.

In the model presented, the average activity of a population is described in terms of an “instantaneous” firing rate that results from a sigmoidal conversion from average membrane depolarization. This quantity will fluctuate at the same time scale of any oscillatory dynamic in the model. Thus, this model is not well suited to answer questions regarding long term changes in rate (over minutes) typically reported in experimental work. Further, the model lacks descriptions of ion channels and neurotransmitters that could permit examinations of the role of nonlinear synaptic integration, or changes in patterns in firing such as bursting etc.

We add to limitations in the discussion:

"Further, abstraction of neuronal activity in the model to a mean instantaneous firing rate, makes it difficult to compare our results to spiking models which include the full complexity of synaptic communication.”

L503-505

Nonetheless, these changes in hyperdirect strength are well justified by experimental evidence [8,9,51] and our model is well suited to explain the counterintuitive effect of how decreased cortical input to the STN can occur simultaneously with enhanced cortical-subthalamic coherence. We posit that this change in timing between cortical and subthalamic populations may be responsible for changes in rhythm:

“…β_1 _rhythms amplified by the STN/GPe loop can return to motor cortex- effectively reinforcing synchrony of the CBGT at this frequency, and potentially explaining how reduced HD transmission in PD can be observed alongside elevated cortico-subthalamic coherence [9]. How this translates to enhanced STN firing seen in primate models [51] is unclear, and would require models incorporating both oscillatory dynamics and detailed synaptic conductances to explain.”

L410-415

We also point the reviewer to our related response regarding 3.12.

2.3. “The nature of simulated stimulation should be clearly stated in the results, to guide the reader regarding the comparability to available treatment strategies. Even after diving into the methods, it was impossible for this reviewer to understand whether DBSlike high-frequency stimulation, single pulse stimulation or oscillatory “currents” or a non-electrical network proxy of stimulation was applied.”

We have expanded on the modelling of stimulation in the results:

“Stimulation was modelled through the addition of a sinusoidal voltage to the average membrane potential of the superficial cell layer in the cortical microcircuit. Input amplitude was fixed to 1/3 of the intrinsic noise level and the central frequency was set to 18 Hz. The phase was constructed to preserve a fixed phase alignment with the bandlimited signal (at β_1_) sensed from the STN population.”

L259-263

We also now discuss the limitations of this approach, and the reasons why more realistic models of DBS were not employed:

“The simplification of dynamics to a single lumped time constant per population can obfuscate the interaction between inputs on distinct time-scales. More realistic models of brain stimulation, such as high frequency (120 Hz) pulse trains for DBS, would require conductance models, in addition to finite element models to capture spatial specificity of a given stimulation modality.”

L499-504

We have also expanded figure 4 to include a schematic of the stimulation setup, indicating the chosen stimulation and sensing sites. 

“Figure 4 – **A model of dual-site controlled stimulation of the motor cortex using signals sensed in the subthalamic nucleus can modulate beta activity across the network. (A)** Schematic for phase specific neuromodulation in the rodent brain. Activity at the STN is used to gate stimulation delivery at M2. (B) Stimulation waveforms were generated using an 18 Hz sinusoid, with phase constructed to be offset against an on-line phase estimate of STN activity. Stimulation was delivered as a direct voltage injection to the stimulated population (superficial layer of M2).“ 

Fig 4. legend

We also point the reviewer to relevant responses 1.8 and 1.9 regarding different sensing/stimulation sites and impact of varying critical stimulation parameters such as threshold and amplitude.

2.4. “Would the stimulation be testable in animal models or is it abstract? To increase the translational value of the submission, I would suggest to elaborate on the available neuroscience methods that could experimentally reproduce the results in animals or humans.”

This is an important point which we now further emphasize in the manuscript:

“To experimentally test our predictions that phase specific stimulation can induce changes in circuit-wide synchronization, it would be necessary to make extensive multi-site recordings during phase locked stimulation. Experimental setups for electrical stimulation have recently been described in 6-OHDA lesioned rats [31] which has the potential to test the PD specific aspects of this work, such as the conditioning role of HD and its potential to be mimicked by stimulation. State dependent effects could be also be tested by inducing pathway specific changes, either via pharmacological, optogenetic [50], or electrical [56] means, and then analysing the effects of phase specific stimulation across a circuit of interest. Recent work outside of the CBGT circuit, has shown that optogenetic stimulation can achieve phase specific modulations of network-wide activity similar to that described here [57].“

L454-462

Further, we detail the translational value of this work and describe how stimulation might be implemented *in vivo*:

“Here we model cortical stimulation, locked to activity sensed in the STN, a configuration chosen as it provided better suppression of STN β_1_ rhythms than the reverse stimulation/sensing combination (see supplementary figure S3). Stimulation was modelled as a sinusoidal input to the superficial pyramidal cells, similar to that resulting from transcranial alternating current stimulation (tACS) which has been shown to entrain sub-threshold cortical activity [58] in a stimulation phase dependent manner [59]. Thus, predictions from this theoretical model could be tested using a combined depth electrode implanted in the STN and closed-loop control of tACS stimulation. It should be noted that stimulation of the STN phase locked to cortical activity can also be used to achieve similar effects if stimulation amplitude and thresholds are optimised according to the cost function of interest (e.g. β_1_ suppression; supplementary figure S4)”

L464-473

2.5. “In a similar vein, given the relevance of the paper to the DBS community the localization of sensing and stimulation for future applications should be stated more clearly. To this reviewer it was hard to understand why cortical instead of subthalamic phase specific stimulation was implemented.”

Cortical stimulation was chosen to be the focus of the paper, as it provided superior suppression of STN beta power compared to STN/M2 stimulation/sensing for the same stimulation thresholds and amplitudes (please see the new supplementary figure S3 and S4 included in this revision) as well as superior recovery of network states supplementary figure S12), a primary goal of this work. We also performed an analysis of state recovery using STN/M2 stimulation/sensing, and found that this setup was not able to flexibly recover the network states in this model. 

We clarify in text and include additional analysis to supplementary information:

“This stimulation/sensing pair was chosen as it provided superior STN beta suppression than the alternative scenario with STN stimulation with cortical sensing for the same stimulation thresholds and amplitudes (supplementary figure S4). Furthermore, supplementary analyses (figure S12)  showed that flexible state recovery (a principle goal of this work; see section Phase Stimulation Can Mimic the Spectral Features…) with STN stimulation was inferior when compared to the cortical stimulation presented.”

L254-259

In light of the new analysis summarised in S4 and S12 and responses 1.8-1.9:

“Despite this, experimental evidence for therapeutic effects of cortical stimulation are inconsistent (see review [60]), yet no closed-loop approaches as presented here have been tested. The dual effects of phase specific stimulation (i.e., achieving both amplification and suppression), combined with recent evidence that cortical stimulation can suppress pathological activity in vivo [50,61] highlight the clinical potential for well-timed cortical stimulation. Nonetheless, our modelling suggested that although STN stimulation paired to cortical sensing exhibited inferior state recovery (supplementary figure S12), it can suppress STN β_1 _(figure S4).”

L474-481

And add corresponding supplementary figure S12:

“S12 Supplementary Figure – State Recovery when Using STN stimulation Phase Locked to Motor Cortical Activity

S12 Supplementary Figure – **Analysis of state recovery when using STN stimulation phase locked to activity sensed in the motor cortex.**  (A, B, and C) Stimulation was applied across 12 phases in the fitted model and data were compared (using pooled R2) to each network state using: the concatenated spectra (D); the matrix of PLV angle/magnitude (E); and the two features combined (F). These results show that spectral fingerprints from stimulation outcomes resembles that from different network states (i.e., a change in synaptic connectivity) depending upon the phase at which stimulation was delivered. (G, H, I) The above was repeated but when varying the connection strength of the PS pathway. Results are plot as a heatmap color-coded to indicate the best fitting state at each phase (x-axis; angle of stimulation relative to STN activity) and connection strength ( y-axis; percentage of fitted synaptic strength, dashed line indicates 100%- i.e., the model fit to the 6-OHDA lesion data and plot in D, E, and F). (D, E, and F) Same as G, H, and I, but for modulations of HD pathway strength.”

SI L210-221

2.6. “Most of the times, stimulation is mentioned without stating stimulation location, which could be confusing to clinicians who expect subthalamic stimulation that is readily testable in patients.”

We now ensure that the respective sensing and stimulation locations are properly declared throughout, and add regular reminders that the stimulation is *cortical* and controlled according to *activity sensed in the STN*.

2.7. “This is aggravated by the introduction that motivates the results section with phase specific DBS. Now cortical stimulation would not be considered deep brain stimulation, but would the authors suggest electrocorticography as a potentially relevant sensing and stimulation approach? This should be discussed.”

We thank the reviewer for highlighting this issue and have now rephrased the introduction to reflect recent work establishing multi-site stimulation control as a precedent for exploring policies using both STN and cortical activity:

“Furthermore, the feasibility of multi-site control policies [31,32] has expanded the potential for phase specific stimulation to manipulate whole networks - with the possibility of delivering stimulation to one region of the brain (e.g., the STN) in closed-loop with that recorded at another (e.g., in the cortex).”

L91-94

And add:

“Specifically, following experimental work establishing dual-site stimulation and sensing [32,34], we explored both STN and motor cortex as potential sites for stimulation control and delivery.”

L105-107

And add in our discussion:

“This setup could be readily implemented with current technology leveraging paired ECoG recordings with DBS [32,34].“

L478-481

2.8. “The authors state in the first paragraph of the results that a qualitative feature of significant feedback of beta oscillations from subcortex to cortex could be produced by the model. To the best of my knowledge, all directionality analyses in human patients have demonstrated that beta activity is driven by cortex (e.g. Litvak et al., 2011).”

This is a feature of the original 6-OHDA lesioned rodent data from which this model is derived (reported in [87]). We add:

“Several qualitative features of the experimental data (reported in [36]) were well reproduced: …); 2) significant STN→M2 directed functional connectivity indicating feedback of beta oscillations^1^”

L132-134

And add the corresponding note:

“Note this feature is pronounced in the original 6-OHDA lesioned rodent data on which this study was based but has not been found in human data where functional connectivity is predominantly lead by cortical activity [37,38]”

L134-138

Note also our response to reviewers comment 1.17 which highlights the potential limitation of investigating data from animals under anaesthetic. We add to discussion:

“… activity recorded under urethane anaesthesia… may overemphasize certain pathways such as thalamocortical feedbacks that exhibit increased strength under anaesthesia [64].”

L510-512

2.9. “The manuscript uses a lot of non-standard abbreviations, making it very hard to read and follow. I would suggest to try and cut down on the number of abbreviations and placeholder terms to improve comprehensibility.”

We remove unnecessary abbreviations – please see also our response to comment 1.35.

3. **Reviewer #3:**

3.1. “**General comments**
This study examines changes in network connectivity and the effect of phase-dependent stimulation in a neural mass model of the cortico-basal ganglia-thalamic circuit. It is concluded that the pattern of beta synchrony in the network depends on connectivity and that well-timed stimulation of the cortex can recover the original state of the network.”

In response to the reviewer’s comments, we have: (1) expanded our limitations segment significantly (c.f., 3.3); (2) made important checks to ensure that the major claims of our study hold given changes in how models were fit (c.f., 3.5); and (3) justifying the existence of bimodal features in our model (c.f., 3.4). Several of this reviewer’s comments overlap with issues raised by R1 and R2, in these cases we refer to the relevant comments, but also reiterate the various changes made to address them.

3.2. “The questions addressed are interesting and topical. However, the message is not very clear. The results are quite long and very discursive, making them difficult to follow or to identify the main findings. In comparison, the Discussion is quite ‘light’ and doesn’t address the nuances of the model or the details of mechanisms involved.”

We have made extensive edits to the results section, to improve the succinctness. In particular we reduce comments or digressions from reporting of the results, preserving the important parts for the now extended discussion. We now discuss comparisons with other models (see comment 1.28 and below), potential experiments to test our hypotheses (see comment 2.4), as well as outlining important limitations following the reviewer’s comments below.

3.3. “A thorough comparison with previous studies is missing and the limitations of the study and model are not addressed.”

We now expand the segment on the study’s limitations, detailing the approximations made (i.e. neural mass models, fitting procedure, and stimulation):

“Neural mass models approximate neuronal dynamics in a way that make them amenable to extensive fitting and simulation, but eschews descriptions of phenomena such as complex neuronal spiking or nonlinear integration [64]. The simplification of dynamics to a single lumped time constant per population can obfuscate the interaction between inputs on distinct time-scales. More realistic models of brain stimulation, such as high frequency (120 Hz) pulse trains for DBS, would require conductance models, in addition to finite element models to capture spatial specificity of a given stimulation modality. Further, abstraction of neuronal activity in the model to a mean instantaneous firing rate, makes it difficult to compare our result to spiking models which can include the full complexity of multiple synaptic conductances.

Moreover, rhythms at high beta/gamma rhythms are abstracted in our model by a bimodal spectrum that results from an inability to capture higher frequency activity thought to be important in prokinetic states [32,36]. The model also omits low frequency alpha/theta which has been implicated in this circuit in dystonia [65] but were not present in the experimental data used to fit the models. Furthermore, models were fit to activity recorded under urethane anaesthesia and may be only qualitatively similar to that seen in the awake animal.  This may overemphasize pathways such as thalamocortical feedbacks that exhibit increased strength under anaesthesia [66]. However, urethane’s effects on neurotransmitter function are small compared to other anaesthetics [67], and dose dependency allows access to cortically activated states which most resembles the waking brain [35].

Model extensions could incorporate additional neuronal populations, such as pallido-striatal arkypallidal cells [68], multiple cortical sources of hyperdirect pathway, distinct populations of striatal medium spiny neurons, or pallidal interneurons. Specifically, inclusion of arkypallidal projections would also allow us to predict how alternative models considering pallido-striatal origins of beta oscillations [69] may alter response to stimulation. These additions to the circuit model would render a larger number of states recoverable by phase locked stimulation, but also demand increased spatial and temporal specificity to access them. Furthermore, the dataset here is limited in its ability to answer these questions due to the paucity of spatial resolution in the cortical recordings (ECoG from a single cortical “screw”).

L497-523

Further, we discuss previous work investigating cortical stimulation for PD:

“Despite this, experimental evidence for therapeutic effects of cortical stimulation are inconsistent (see review [60]), yet no closed-loop approaches as presented here have been tested. The dual effects of phase specific stimulation (i.e., achieving both amplification and suppression), combined with recent evidence that cortical stimulation can suppress pathological activity in vivo [50,61] highlight the clinical potential for well-timed cortical stimulation. Nonetheless, our modelling suggested that although STN stimulation paired to cortical sensing exhibited inferior state recovery (supplementary figure S12), it can suppress STN β1 (figure S4). This setup could be readily implemented with current technology leveraging paired ECoG recordings with DBS [32,34].”

L474-481

3.4. “There is a question around the bimodal distribution of the power spectrum of the simulated neural field data. This bimodal distribution does not appear to be present in the experimental data on which the model is based and constrained. When introduced in the results the bimodal distribution is linked to other experimentally reported data, but how does this fit with the data on which the model has been based, and why are there differences between the two?”

We now include new analysis exploring the bimodal nature of the experimental spectral features (please see response to comment 1.15). This analysis highlights that bimodal beta band features exist in not only the group averaged data, but also those derived from individual recordings. This bimodality is not present in the spectra -but is present in the directed functional connectivity. The fact that this has resulted in a bimodal power spectrum in our model is discussed in our response to comment 3.6 below.

We add to SI (figure in comment 1.15):

“S2 Supplementary Figure – Analysis of multi-Gaussian fits to empirical data”

SI L11-18

And results:

“The model also produced activity at two sub-peaks at lower (β1: 14-21 Hz) and upper (β2: 21-30 Hz) beta frequencies, reflecting the bimodal structure of directed functional connectivity (see analysis of multiple Gaussian fits to features from individual animals; supplementary information S2) …”

L141-144

3.5. “Also, why did the optimization of model parameters converge on this model which appears different to the experimental data, does the model still represent the experimental data/states?”

The model fit shown in the paper was optimized using the mean squared error, which due to the difference between the scales of the auto-spectra (normalized to unit variance) and functional connectivity features (bounded [0 1]) had the effect of biasing fits to more accurately recapitulate the directed functional connectivity at the expense of fits to the auto-spectra- see table below and updated supporting figure 1.

|  | Coefficient of determination (R^2^) – *median ±IQR* |
| --- | --- |
| Auto-spectra | -1.22±0.37 |
| Directed functional connectivity | 0.23±0.60 |
| Pooled | 0.14±1.43 |

As an alternative model, we performed a second model fit using the pooled R^2^ as a cost function. We found that this model reproduced the auto-spectra more accurately, but at the expense of recapitulating functional connectivity. Importantly, we show that two main findings (see figure 4 with this alternative model below) of this paper are robust to this change in model: (A) β_1_ suppression by phase locked stimulation; (B) state recovery of HD-Up/Down exhibits similar pattern to those seen in the presented model.

This alternative model, parametrised to emphasize auto-spectra, exhibited: (A) phase specific modulation of beta rhythms; (B) antiphase modulation of β_1 _ and β_2 _power and coherence; (C) and state recovery of HD-Up and HD-Down, similar to that seen in the main reported model.

Please see also our response to comment 1.15 regarding specific degrees of fit.

3.6. “The type of model used, i.e., a neural mass model, is not described until page 2 of the Methods. This should be clarified in the Abstract, Introduction and Results.”

We thank the referee for raising this point and now better describe our modelling approach in the sections highlighted by the reviewer, as well as discuss this model’s limitations.

We add to abstract:

“…we setup a neural mass model of population activity”

L26

And to the introduction:

“…we used a neural mass model of the CBGT circuit…”

L97

And to the results:

“We simulated a neural mass model of neural population activity…”

L112

“We constrained the parameters of a neural mass model of the CBGT circuit…”

L125

“Since the parameters of the neural mass model were constrained using …”

L157

3.7. “Similarly it should be clarified here that the spectral ‘fingerprints’ referred to are at the population level.”

We agree that this is important to state, and now clarify that “fingerprints” summarise the pattern of activity and synchronization of population activity across the model. We better define from the introduction:

“We summarise the changes in neural population activity (i.e., change to spectra and functional connectivity) associated with different network states (i.e., changes to structural connectivity) using statistical summaries that we term *spectral fingerprints* [33][18]….”

L98-101

And modify in the results:

“…we compared the spectral fingerprints of spontaneous population activity following alterations of synaptic connectivity…”

L309-310

3.8. “**Specific comments**
What is the motivation for targeting the cortex for stimulation? This should be outlined in the Introduction.”

In response to the reviewers comments we have tried both stimulation/sensing pairings for the STN and cortex (see also our response to 1.27 and 2.5). We present cortical stimulation as it was most effective at suppressing STN beta rhythms at a given stimulation threshold and amplitude:

“This stimulation/sensing pair was chosen as it provided superior STN beta suppression than the alternative scenario with STN stimulation with cortical sensing for the same stimulation thresholds and amplitudes (supplementary figure S4). Furthermore, supplementary analyses (figure S12) showed that flexible state recovery (a principle goal of this work; see section Phase Stimulation Can Mimic the Spectral Features…) with STN stimulation was inferior when compared to the cortical stimulation presented.”

L254-259

We also now introduce some experimental context for why we investigated these pairings:

“Furthermore, the feasibility of multi-site control policies [31,32] has expanded the potential for phase specific stimulation to manipulate whole networks - with the possibility of delivering stimulation to one region of the brain (e.g., the STN) in closed-loop with that recorded at another (e.g., in the cortex).”

L91-94

“Specifically, following experimental work establishing dual-site stimulation and sensing [32,34], we explored both STN and motor cortex as potential sites for stimulation control and delivery.”

L105-107

But add to discussion the caveat that whist STN stimulation can be effective at suppressing beta given the right parameters, we found that recovery of network states was comparatively poor:

“Nonetheless, our modelling suggested that although STN stimulation paired with cortical sensing exhibited inferior state recovery (supplementary figure S12), it can suppress STN β_1 _(figure S4). This setup could be readily implemented with current technology leveraging ECoG recordings during DBS [32,34].”

L478-481

3.9. “GPe-GPe interconnectivity is not considered in the model. How would this impact the results?”

We add to our limitations how structural changes to the model would be expected to affect our results:

“Model extensions could incorporate additional neuronal populations, such as pallido-striatal arkypallidal cells [22], multiple cortical sources of hyperdirect pathway, distinct populations of striatal medium spiny neurons, or pallidal interneurons.. These additions to the circuit model would render a larger number of states recoverable by phase locked stimulation, but also demand increased spatial and temporal specificity to access them.”

L515-523

3.10. “The model describes well the power spectrum of the population of neurons and directed functional connectivity within the network. However, is this is a true representation of the network state as the behavior of individual neurons is not captured?”

We add this important consideration to our limitations of the model – namely the ability for the model to capture dynamics beyond the average depolarization seen at the population level. 

“Neural mass models approximate neuronal dynamics in a way that make them amenable to extensive fitting and simulation, but eschews descriptions of phenomena such as complex neuronal spiking or nonlinear integration [64]. The simplification of dynamics to a single lumped time constant per population can obfuscate the interaction between inputs on distinct time-scales.”

L497-505

3.11. “Figure 1 and 2 indicate connections from the secondary motor cortex (M2) only to STN. However, the STN receives projections from the primary motor cortex (MI), the supplementary motor area (SMA), and premotor cortex (e.g. Nambu et al.). Is this a reasonable assumption in the model?”

Indeed, inclusion of these structural additions to the model would be very valuable. However, we note the limitations of the available data- namely the poor spatial resolution afforded by our cortical ECoG and add this as a consideration to our discussion:

" Furthermore, the dataset here is limited in its ability to answer these questions due to the paucity of spatial resolution in the cortical recordings (ECoG from a single cortical “screw”).”

L521-523

3.12. “Down regulation of the hyperdirect pathway is an assumed condition of the Parkinsonian/dopamine depleted state (p. 24) based on references [9-11]. How does this fit with the conclusions of increased effective connectivity in the hyperdirect pathway obtained using similar Bayesian modelling methods in the same data set in the model referred to in [66]?”

This is an important point to reconcile. We add:

“In agreement with other models exploring thalamocortical feedback [13], we suggest that β1 rhythms amplified by the STN/GPe loop can return to motor cortex- effectively reinforcing synchrony across the CBGT at this frequency. This potentially explains how reduced HD transmission in PD can be observed alongside elevated cortico-subthalamic coherence [9]. How this translates to of enhanced an STN firing rate seen in primate models [51] is unclear, and would require models incorporating both oscillatory dynamics and detailed synaptic conductances to explain. Our model is distinct from a previous dynamic causal modelling study in which effective connectivity of the HD pathway was found to be raised in the 6-OHDA state [49], a finding in disagreement with findings of impaired cortico-subthalamic efficacy [8,9], and likely arising due to the relative insignificance of the STN/GPe circuit in that model”

L409-419

Furthermore, see related comment 2.2.

3.13. “What happens when the HD pathway strength is decreased and PS strength is simultaneously increased? Why were changes in the hyperdirect and pallido-subthalamic connection strengths examined independently, rather than allowing simultaneous changes in both? This would seem to be a better representation of dopamine depletion/ worsening symptoms then changing them independently.”

This composite change is reflected in the configuration of the fitted model that was constrained to data from the 6-OHDA lesioned state. We change the HD and PS connections individually in order to isolate their effects upon both network connectivity and spectral features of the model. Please see also comment 1.4 in which we make changes to remind the reader of the basis of the fitted model.

Furthermore, in this revision we have also included a set of empirical network states (i.e., those derived from the experimental 6-OHDA lesioned or control data) in which both pathways (as well as others) are expected to be modulated (see also response to comment 1.2):

“…These data reflect not only hypothesised changes in HD and PS pathways, but reconfigurations of the wider network expected to accompany dopamine depletion.”

L344-346

Please see also our response to comment 1.2.

3.14. “Why was a sinusoidal stimulation chosen for the cortical stimulation? What was the frequency of this stimulation? This is obviously different to the type of stimulation that is delivered clinically.”

We chose to use sinusoidal stimulation, as this allowed us to provide a modulation at the same time-scale as intrinsic activity. As discussed in the extended limitations outlined in response 3.4, the nature of the neural mass formulation does not suit itself to modelling stimulation modalities used clinically. 

With regards to details of the stimulation please see also comments 2.3 and 2.4. We make clear in the text that stimulation was delivered at 18 Hz to target β_1_ rhythms.

3.15. “The maximum suppression of beta power provided by the phase locked stimulation seems quite modest (at -30 % in the STN and -17 % in M2). In contrast, the phase locked stimulation was much more effective in increasing beta band power (up to 155 % and 240 % of the baseline value). What is the reason for this ‘asymmetric’ response?”

We agree that the stimulation effect is modest, and detail why this control policy was chosen in a previous comment- please see our response to comment 1.8.

In terms of the asymmetric response – this is a phenomenon that has been observed in previous phase-specific stimulation studies e.g., [26,27], but is not well explained. We add to our discussion of the results:

“We also found that stimulation more readily amplified than suppressed the target rhythm, an asymmetry likely arising from: (A) the maximum limits of suppression (-100%) vs amplification (unbounded); (B) the ability to entrain the system with random inputs which is easier to achieve that providing a precise perturbation to completely suppress an oscillation.”

L433-437
