# Supplementary material for: Stimulating at the right time to recover network states in a model of the cortico-basal ganglia-thalamic circuit
Source: PLoS Comput Biol. 2022 Mar 4;18(3):e1009887. doi: 10.1371/journal.pcbi.1009887 (PMC8939795; doi:10.1371/journal.pcbi.1009887)
Supplement: Supplementary file 4 [file pcbi.1009887.s014.docx]

PCOMPBIOL-D-21-01076

**Stimulating at the Right Time to Recover Network States in a Model of the Cortico-Basal Ganglia-Thalamic Circuit.**

*Timothy O. West, Peter J. Magill, Andrew Sharott, Vladimir Litvak, Simon F. Farmer, Hayriye Cagnan*

Please note, we now refer to the Hagai Bergman’s recent work on the relationship between beta band oscillations and dopaminergic tone:

“In agreement with the model presented here, recent experimental evidence suggests that the specific phase and frequency of beta expression is modulated by changes in dopaminergic tone [49].”

L401-403

**Reviewer #1:**

1.1. “The authors are to be commended for the clear and comprehensive response to the prior review, and their extensive revision of what is now a much more clear and compelling manuscript. Nonetheless several important points remain to be addressed.”

We thank the reviewer for their careful reading of our manuscript and our responses. Their contributions have undoubtedly improved the quality of the work. We address their remaining concerns below.

1.2. “An important remaining limitation is the absence of comparisons to clinical standard high frequency (130 Hz) STN DBS. The comparisons to other (quite low frequency modalities) is a step in the right direction, but, given the added complexity of implementing the proposed phase-specific stimulation, a comparison to the remarkably simple open loop 130 Hz is required.”

We agree that this is an important consideration. Accordingly, we have attempted several approaches to incorporate high frequency stimulation.

First, we injected an input reflecting open-loop high frequency stimulation, and simulated the response of the STN with (blue) or without stimulation (orange). The stimulation waveform comprised either 1ms wide monophasic pulsatile input (A and C), or continuous sinusoid (B and D). In both cases, input entrained local dynamics at the stimulation frequency (i.e. 130 Hz), but cross-frequency effects (i.e., attenuation of beta band activity) were not observed, even at very large stimulation amplitudes (in which there was a broadband increase in activity; data not shown). It should be noted that entrainment of the subthalamic population to the stimulation frequency (i.e., 130 Hz) was not propagated to other nodes and therefore the stimulation was effectively filtered by the network.

Previous work, utilising a neural mass model of the cortico-basal ganglia, has captured the effect of high frequency stimulation by integrating stimulation pulses and scaling the total charge by the membrane capacitance to yield the voltage change per integration time step (every 1 ms), which was then directly injected into the target population (Grado et al 2018). An alternative approach would be to model the effect of high frequency stimulation as a switch from bursting at low frequency rhythms, to high frequency and tonic firing (Rubin and Terman, 2004). These effects can be captured in our neural mass model by biasing the firing rate of the target population via modification of the sigmoid transfer function to include a baseline offset. This would reflect an increased overall activation of the target population at lower membrane potentials. This is indeed sufficient to attenuate beta rhythms in our model (see below):

Please note that in panel (A) above, firing rate represents a deviation from a background rate of firing, where a negative change means that the population will fire less than the average. Thus the offset in the sigmoid (orange curve) biases the node towards higher firing rates at smaller membrane potentials. 

While our model can capture various mechanisms proposed to underlie high frequency stimulation, in the absence of a model that can operate at a wide range of time scales (e.g. spiking or conductance based models), it is not feasible to make a direct comparison with phase locked stimulation.

Accordingly we now detail these findings in our limitations section

“Neural mass models simplify neuronal dynamics to a formalism that makes them amenable to parameter optimisation and intensive simulation, however the approximations made in the process make them ill equipped to investigate phenomena such as complex spiking and nonlinear integration of inputs. Whilst in this model it is not possible to directly simulate high frequency stimulation, it is possible to either enact a constant voltage change [62] or bias the sigmoid transfer function to favour tonic activation, a mechanism proposed by the model of Rubin and Terman [66]. Despite these limits in directly modelling high frequency stimulation, our model does however capture experimental features of phase specific stimulation at beta frequencies such as the emergence of secondary rhythms [26] and phase dependent modulation of cortical rhythms [30].”

L502-510

1.3. “Further, several elements of the comparison of stimulation modalities (S3) are puzzling and require some further explanation. It is unclear in S3 why the power in the 0% condition with the phase specific stimulation is approximately twice as large as the baseline power in the other conditions that were tested?” 

We thank the referee for pointing out this issue. This difference in baseline power was due to an error in the coding used to normalise the spectra. We now correct the figures to show their unnormalized values. These values are roughly equal across all stimulation modalities (figures 2, 4 and S3). Differences that remain in baseline power between playback and phase specific stimulation are linked to the same sections of data being stimulated in the playback trials as the original phase specific stimulation. Since these did not necessarily correspond to periods of high power for playback, the baseline in this condition is slightly less.

1.4. “And not clear how this power spectra compares to those in Figure 2 or the changes in beta power in Figure 4B, where the increases in beta power at 330 degrees are much more than +42%, and there certainly does not look to be a ~50% increase in beta2 power?”

We now remove the normalization and unify power calculations to ensure that spectra are comparable across figures. Thus all baseline beta peaks match between figures. 

With respect to differences in the modulations, these resulted from different methods being used to compute power– in figure 4B we look specifically at periods of the simulation in which stimulation was delivered, whereas previously in S3 we measured changes based on the whole simulated trace. Again, we unify analyses to look exclusively at the periods of the data during which stimulation was delivered. We clarify in methods:

“ARCs were constructed from epochs of data during which stimulation was delivered. Epochs were matched according to stimulation duration when comparing ARCs and the unstimulated model.”

L815-817

1.5. “The method relies on estimation of the phase of noisy signals and some quantitative evaluation of the effects of errors in phase estimation, as will occur with low SNR signals, in vivo, especially, when stimulation is effective at reduction of beta1 activity in STN. This will help to set specifications for implementation of the proposed approach.”

We now perform an analysis of the amplitude response characteristics of phase locked stimulation under modulation of the signal to noise ratio of the sensing signal. This is now enclosed in the supplementary materials:

“We also estimated the effect of varying signal-to-noise ratio (measured as SNR dB) upon the phase locking algorithm. We first used a noisy sinusoid signal (described above) and added Gaussian white noise as additive noise to modulate the SNR. Noise was given with a specific ratio with respect to the variance of the signal in order to achieve a range of observation scenarios from -20 dB to +10 dB. Again, we assess accuracy of the on-line phase estimate with respect to phase estimated from the Hilbert Transform, using the PLV.

The results shown in the figure below indicate that on-line phase estimates are stable in the range -5dB to +10 dB (i.e., good signal quality). Below -10dB, errors in phase estimation introduced by observation noise confound the ability of phase specific stimulation to achieve suppressive effects on beta rhythms. Further, at very low SNR (<-15 dB) phase locked stimulation has an entrainment effect causing exclusively amplifying effects on beta power. 

Figure – Effect of SNR upon on-line phase locking algorithm (zero-crossing detection and interpolation). (A) Effects of SNR on accuracy of phase estimate in noisy sine model, using the PLV to compare against the Hilbert phase estimate. (B and C) Effects of SNR on the ability of phase specific stimulation to modulate STN beta rhythms in the CBGT model.”

SI L208-221

These findings indicate that in real world settings, more sophisticated phase-estimation algorithms are likely to be required. We add to the discussion:

“ We also note the dependency between phase specific stimulation and the signal quality at the sensing electrode. Our supplementary analyses (see appendix S10) show that the zero-crossing technique used for phase detection is only effective at suppressing beta frequency activity up to around -5dB (~1:3 SNR). Real world applications will likely require more refined phase estimation techniques [63], especially those making explicit estimation of observation noise [64].”

L489-493

1.6. “The Limitations in the Discussion should consider those associated with the direct “intracellular” stimulation of a select subset of modeled cortical neurons and how this mifght compare to electric epicortical stimulation.”

This is an important consideration, we add to the discussion:

“Finally, the model of stimulation presented here does not incorporate the spatial complexity of real life epicortical stimulation, rather we model the stimulation as current injection directly to a single neuronal population. Applications of this technology in vivo would require detailed understanding of the spatial extent of stimulation to understand how electrical spread could affect pathway specific activity.”

L529-532

1.7. “The variance accounted for metric in Fig 6A is puzzling as it is not clear how R^2 can either take on negative values or have absolute values greater than 1.”

With respect to how R^2^ can be negative, we refer to the calculation of R^2^ as equal to “1-(Residual Sum of Squares/Total Sum of Squares). In the case where RSS>TSS (and so R^2^<0), then this simply means that the fit is worse than a straight line going through the mean ($y= \bar{x}$). Because of this equation- R^2^ is bounded [-inf 1]. We add to methods:

“$R_{pooled}^{2}$ varies between -∞ and 1, with 1 indicating a perfect fit, and negative coefficients indicating that the average fit is worse than that of a straight line going through the mean.”

L833-835

With respect to values of R^2^ greater than 1, we cannot see these in any of the figures. It may be the case that the minus signs are too small, and so we increase the inset font size of S1 to make this clearer.

Following consideration of R1’s concerns raised here, we have removed analysis of the angle of the phase locking values when computing network fingerprints. This is because the circular R^2^ , used for this factor, was bounded [0 1] and therefore introduced a bias (i.e., artificially inflated very bad models). This alteration impacts figures 5 and supplementary figure 6 (see annotated changes below), but does not cause any change to the conclusions that may be drawn from them. 

1.8. “Color coding of spectra in bottom panel of Fig 5B looks swapped as power in STN with max B1 amplifying (blue) is greater than power with max B1 suppressive (orange)”

We thank the reviewer for spotting this error, which is now corrected in the revised figure.

1.9. “Sanabria et al. stimulated GPi not STN.”

Thank you for pointing this out. We now correct this:

“In Parkinsonism specifically, stimulation of the basal ganglia (such as the subthalamic nucleus STN, or internal globus pallidus GPi) has been shown to both amplify or suppress pathological beta oscillations, dependent on the specific phase targeted in both primate disease models [26] and patients [25].”

L89-92

“…reported by Sanabria and colleagues, in which phase locked stimulation of GPi…”

L446

1.10. Annotated Changes to figure 5

1.11. Annotated changes to supplementary figure 6
